# Global conformations of *Pichia pastoris* complex I are distinguished by the binding of a unique interdomain bridging subunit

**DOI:** 10.1126/sciadv.adz0693

**Published:** 2025-10-01

**Authors:** Chris Seunggyu Lee, Daniel N. Grba, John J. Wright, Bozhidar S. Ivanov, Judy Hirst

**Affiliations:** The Medical Research Council Mitochondrial Biology Unit, University of Cambridge, Keith Peters Building, Cambridge Biomedical Campus, Hills Road, Cambridge CB2 0XY, UK.

## Abstract

Complex I (CI; NADH ubiquinone oxidoreductase) is central to energy generation and metabolic homeostasis in mammalian cells but contributes to adverse outcome pathways under challenging conditions. During ischemia, mammalian CI transitions from a turnover-ready, structurally “closed” state toward a dormant “open” state that prevents it from functioning in reverse during reperfusion to produce reactive oxygen species. Unfortunately, simpler, genetically tractable CI models do not recapitulate the same regulatory behavior, compromising mechanistic studies. Here, we report the structure of isolated CI from the yeast *Pichia pastoris* (*Pp*-CI) and identify distinct closed and open states that resemble those of mammalian CI. Notably, a hitherto-unknown protein (NUQM) completes an interdomain bridge in only the closed state, implying that NUQM stabilizes it by restricting the conformational changes of opening. The direct correlation of NUQM binding with closed/open status in *Pp*-CI provides opportunities for investigating regulatory mechanisms relevant to reversible catalysis and ischemia-reperfusion injury.

## Teaser

Cryo-EM structures of complex I from *Pichia pastoris* reveal two conformations differentiated by binding an atypical subunit.

## INTRODUCTION

Mitochondrial NADH [reduced form of NAD^+^ (nicotinamide adenine dinucleotide)]:ubiquinone oxidoreductase [complex I (CI)] is a large multisubunit enzyme located in the inner mitochondrial membrane where, together with the other electron transport chain complexes, it maintains the proton-motive force and the NADH/NAD^+^ redox balance that are required for adenosine 5′-triphosphate generation and a host of metabolic processes (*1*–*3*). As a result of these central roles, CI dysfunctions result in numerous metabolic and neuromuscular diseases (*4*–*7*) with CI regulation and activity relevant in pathophysiological scenarios that occur at the extremes of homeostasis, such as ischemia-reperfusion (IR) injury in cardiac arrests and strokes (*8*, *9*).

**Fig. 1.**
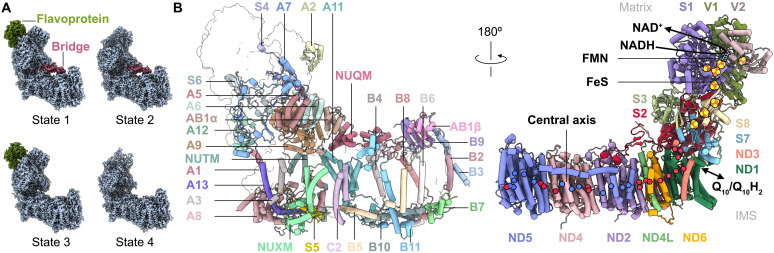
Structure of CI from *Pp* mitochondria. (**A**) Cryo-EM densities for the four observed states of *Pp*-CI. Regions of compositional heterogeneity are labeled and colored. (**B**) Subunit composition of *Pp*-CI showing the supernumerary subunits (left, core subunits in outline) responsible for assembly and stability and the core subunits (right) responsible for enzyme catalysis. Key elements are labeled in black, with red and blue circles indicating the Cα positions of conserved acidic and basic residues that are implicated in proton transfer across the membrane and that connect the membrane domain to the Q_10_-binding site. The matrix and intermembrane space (IMS) sides are indicated. Sequence names for nuclear-encoded subunits conserved in human CI have been abbreviated for clarity (for example, NDUFS4 to S4). Q_10_H_2_, reduced Q_10_.

The process catalyzed by CI comprises a redox reaction coupled to proton translocation across the inner mitochondrial membrane. In the hydrophilic domain, two electrons are passed from NADH to ubiquinone [typically ubiquinone-10 (Q_10_)] via a flavin mononucleotide (FMN) and an extended chain of iron-sulfur (FeS) clusters (Fig. 1), forming ubiquinol, which is reoxidized to sustain the electron transport chain and reduce O_2_ to H_2_O. The ubiquinone headgroup binds at the top of a long, hydrophobic channel that reaches up toward the FeS clusters from the membrane at the domain interface. Key structural elements from both domains connect together, and redox catalysis meets a chain of conserved charged residues (the E-channel) that leads down into the membrane plane. The energy generated by ubiquinone reduction is captured to drive the translocation of four protons across the transmembrane domain, relayed and controlled by conserved residues and intercalated water molecules (*10*–*15*), to power oxidative phosphorylation (*16*, *17*). However, the choreography of ubiquinone reduction and the mechanism of how the energy is captured, coupled, and transferred by the protein to drive distant reactions, with such high specificity and efficiency, remain under a heavy debate (*12*, *13*, *18*, *19*).

Mammalian CI is composed of 45 subunits, of which 14 are the core subunits conserved across species and 31 are “supernumerary” subunits that vary considerably. Whereas 38 subunits (seven core subunits and all the supernumeraries) are nuclear encoded, the seven core subunits that form the transmembrane domain are encoded in the mitochondrial genome, making them hard to study by mutagenesis. Different model systems are therefore being exploited to aid investigations of different aspects of CI function. Bacterial model systems, such as *Escherichia coli* and *Paracoccus denitrificans*, contain simplified versions of the enzyme (no more than a handful of supernumerary subunits) in their periplasmic membranes (*20*–*23*). These systems are particularly powerful because they enable mutagenesis throughout the core complex, so have been key to the identification of conserved, mechanistically essential residues. However, they are unable to provide insights on the higher-order biochemistry of the mammalian enzyme, including its assembly, regulation, and response to physiological changes such as tissue ischemia, or the roles of its many supernumerary subunits (*7*, *24*–*27*). A range of eukaryotic model systems, from single-cell fungi to mouse, has also been developed for mutagenesis, with the former resting on higher-throughput mutational analyses in the nuclear genome and the latter on the greater biochemical relevance of studying the enzyme in mammalian tissues (*28*–*33*). *Yarrowia lipolytica*, with 28 supernumerary subunits, has been the main fungal model used in structure-function analyses of CI in recent years, with its genetic tractability supporting residue mapping around the ubiquinone-binding site, as well as the roles of residues and subunits in assembly and disease (*28*, *30*, *34*–*36*). However, *Y. lipolytica* CI does not cleanly recapitulate key functional features of the mammalian complex (*37*), including the regulatory transition that converts it from a turnover-ready (structurally closed) resting state to a dormant (structurally open) resting state during mammalian tissue ischemia. The transition is protective against IR injury, because only the closed form is able to catalyze reverse electron transfer (RET) (*38*, *39*) upon reperfusion, resulting in a burst of reactive oxygen species production that can lead to oxidative tissue damage (*31*, *40*–*43*). *Y. lipolytica* CI exhibits a single resting state with mixed characteristics from both the mammalian closed and open states and has not been demonstrated to catalyze in reverse (*38*). Therefore, a genetically tractable simple eukaryotic model system that captures both of these key functional aspects is needed to enable genetic approaches to be deployed to investigate both the regulation and reversibility of the mammalian complex.

Here, we present the structure of CI from the yeast *Pichia pastoris* (*Pp*) [*Pp*-CI; syn. *Komagataella* ssp. (*44*, *45*)]. *Pp* is a methylotrophic yeast—it is capable of respiring on methanol alone—that is highly genetically tractable (*46*). We have previously purified highly active CI from *Pp* mitochondria, reported its subunit composition, and demonstrated that *Pp*-CI is a catalytically reversible enzyme capable of performing RET (*38*, *47*, *48*). Preliminary biochemical experiments to test the proportion of closed and open states in *Pp*-CI revealed a mixture, similar to the mammalian complex, suggesting the possibility of analogous regulatory behavior. Accordingly, our structural analyses now reveal that *Pp*-CI exists in two distinct global states but with the unexpected twist that a hitherto-unknown protein is observed influencing their distribution: Being specifically bound to the closed state, it appears to stabilize it by forming a “bridge” between the two enzyme domains. Our structures provide a rational explanation for the ability of *Pp* (but not *Y. lipolytica*) CI to catalyze RET and demonstrate *Pp*-CI as a comprehensive platform suitable for structure-guided genetic investigations of the regulation of the eukaryotic complex, its canonical resting states, and the mechanisms of CI dysregulation in mammalian pathologies (*9*, *39*, *40*, *42*).

## RESULTS

### Catalytically active *Pp-*CI in Q_10_-containing nanodiscs

Highly active CI was purified from *Pp* cells using lauryl maltose neopentyl glycol (LMNG) as the detergent (fig. S1). Whereas the detergent dodecyl-β-d-maltoside (DDM) that we used previously (*47*) has been observed in the catalytic Q_10_-binding site in mitochondrial CI structures, the bulkier LMNG is expected to be obstructed from entering and has not been observed bound there in any structure so far (*10*, *12*, *49*, *50*). To provide *Pp*-CI with a native-like environment, it was then reconstituted into cMSP26 (circularized membrane scaffold protein of 26 nm in diameter) nanodiscs (NDs) containing phospholipids and Q_10_. The cMSP26 NDs are larger in diameter than the MSP2N2 NDs (~17-nm diameter) used previously, which wrapped too tightly around the similarly sized mammalian enzyme and restricted turnover (*49*). *Pp*-CI in cMSP26 NDs showed high rates of CI catalysis upon addition of the membrane-peripheral “alternative” quinol oxidase (AOX); the rates did not increase substantially when the relatively hydrophilic Q_10_ analog decylubiquinone (DQ) was added, confirming the rapid redox-cycling turnover of the Q_10_ contained in the ND bilayer (fig. S2).

### Structural analyses of *Pp*-CI-NDs

The *Pp*-CI-NDs were frozen onto 0.6/1 holey carbon cryo–electron microscopy (cryo-EM) grids and imaged using a Titan Krios 300-kV microscope for single-particle analysis (figs. S3 and S4 and table S1). During analysis, it quickly became apparent that the sample was compositionally heterogeneous, with some particles lacking the FMN-containing flavoprotein subcomplex (NDUFV1 and NDUFV2, responsible for NADH oxidation) and some particles exhibiting an unexpected density that formed a bridge between the two major arms of the L-shaped enzyme. Thus, targeted particle classification resulted in four states of *Pp*-CI-ND: state 1 (with a flavoprotein and with a bridge), state 2 (without a flavoprotein and with a bridge), state 3 (with a flavoprotein and without a bridge), and state 4 (without a flavoprotein and without a bridge) (Fig. 1A and fig. S3).

To create a model for *Pp*-CI containing both the flavoprotein and bridge, we used only particles containing all the enzyme components (state 1) and ran focused refinements in three subregions to improve the local map quality and create an overall-improved composite map (fig. S5A) before implementing the automated model-building software ModelAngelo (*51*) with known *Pp*-CI subunit sequences (*48*). The resulting model contained all the known subunits but left three further subunit densities unaccounted for. However, the ModelAngelo-predicted sequences for these densities provided protein homology search inputs that were successfully used to identify them (fig. S5B). Two of the subunits are *Pp*-CI structural homologs to known mammalian and yeast CI subunits NDUFB2/NIGM and NDUFC2/NEBM (accession codes CAH2448636 and XP_002492753). The third (XP_002490382) is the subunit that forms the protein bridge; it belongs to the GatB (glutamyl-tRNA amidotransferase subunit B)/YqeY protein superfamily and was identified in earlier *Pp*-CI mass spectrometry analyses but assumed to be an impurity (*48*). The model for the complete (state 1) form of *Pp*-CI-ND, containing a total of 44 subunits (Fig. 1B), was then used as a starting model for the other three states that lacked specific subunits (see Materials and Methods, fig. S6, and tables S2 to S5). The bridge subunit was found to affect the global conformation of the enzyme, with the presence of the bridge resulting in a smaller apparent angle between the two major enzyme domains that is often described as a more “closed” conformation (fig. S7A). In contrast, the presence/absence of the flavoprotein appeared not to cause any other changes, so it is likely unstable and simply prone to dissociation during purification, reconstitution, and/or grid freezing. Although no isolated flavoprotein was detected during data processing, isolated hydrophilic domains were detected and classified into a minor population (fig. S7B). Our further analyses thus focus on states 1 and 3 (which both contain the flavoprotein subcomplex, with and without the bridge subunit, respectively) that refined to final resolutions of 2.8 and 3.3 Å, respectively (Fig. 1A).

### The 44 subunits of *Pp*-CI

Of the 44 subunits of *Pp*-CI, 14 are the conserved core subunits present in all CI structures and sufficient for redox-coupled proton translocation. As expected, these are canonical CI subunits that contain the FMN in the active site for NADH oxidation, eight FeS clusters, and conserved residues that are known to be mechanistically essential, including in the Q_10_-binding site, in the E-channel, and along the central membrane axis. This conserved catalytic machinery is summarized in Fig. 1B but is not the focus of our current investigation. As in other yeast and mammalian species, the core NDUFS2 subunit contains a conserved posttranslationally modified dimethylated arginine (*48*, *52*). Despite the presence of Q_10_ in the bilayer of the *Pp*-CI-NDs, no clear continuous cryo-EM density for it was detected in the binding channel. In total, 28 of the *Pp*-CI supernumerary subunits are homologous to subunits observed previously in structures of CI from other yeast and mammalian species, with the expected NADPH [reduced form of NADP^+^ (nicotinamide adenine dinucleotide phosphate)] bound in NDUFA9 and acylated pantetheine-4′-phosphates on the acyl carrier ACPM1/2 subunits (*48*). The supernumerary bridge subunit, which we name NUQM, and the subunit NUTM detected previously by mass spectrometry (*48*) are unique to the *Pp*-CI structure. These yeast-specific subunit names will be used alongside the human nomenclature for conserved subunits.

**Fig. 2.**
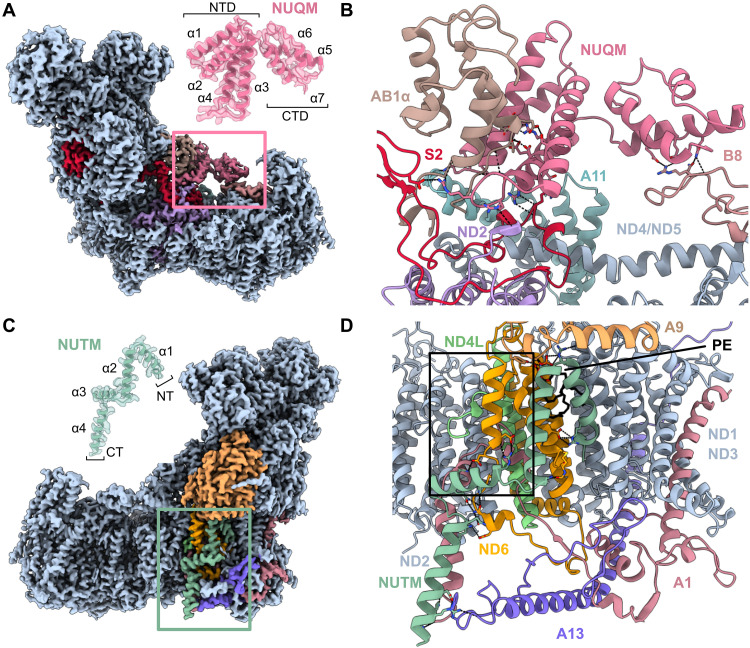
Specific subunits of *Pp*-CI. (**A**) Cryo-EM density of *Pp*-CI highlighting (pink square) the position of NUQM (map and model, inset). (**B**) The interactions of NUQM are dominated by the N-terminal domain (NTD); hydrogen bonds are shown in stick representation. (**C**) Cryo-EM density of *Pp*-CI highlighting (green square) the position of NUTM (map and model, inset). (**D**) Interactions of NUTM with hydrogen bonds are shown in stick representation. The boxed outline highlights the area in which the missing TMH3^ND4L^ and truncated TMH4^ND6^ are compensated for by the NDUFA1 extension. CTD, C-terminal domain. Sequence names for nuclear-encoded subunits conserved in human CI have been abbreviated for clarity (for example, NDUFS2 to S2). α values on insets indicate successive [N-terminal (NT) to C-terminal (CT)] α helices.

NUQM is composed of two domains: Its N-terminal four-helix bundle makes contacts with subunits ACPM and NDUFS2 in the hydrophilic domain and ND2 and NDUFA11 in the membrane domain, completing a multisubunit bridge between the two core domains, whereas its C-terminal three-helix bundle forms only minor contacts with NDUFB8 (Fig. 2, A and B). NUQM is structurally homologous to two proteins of unknown function in the pathogens *Campylobacter jejuni* and *Vibrio parahaemolyticus* (*53*) and to noncatalytic subdomains of GlnRS (tRNA-interacting glutaminyl-tRNA synthetase) and GatB (*54*, *55*).

In NUTM, two transmembrane helices (TMHs) are followed by an amphipathic helix that rests on the bilayer surface and another that extends into the intermembrane space (Fig. 2C). NUTM is unusual in that it occupies a cleft between core membrane subunits ND1/ND3 and ND4L/ND6 underneath the lipid-binding supernumerary subunit NDUFA9, which is otherwise occupied by tightly bound lipids in a variety of species (fig. S8A) (*39*, *56*–*58*). A single lipid is sandwiched between NUTM and the core subunits, isolated from the bulk membrane. Further divergence in this region is present in ND4L, which lacks the otherwise-conserved third TMH, and in the truncated TMH4 of ND6. Both of these changes are compensated by an extension to NDUFA1 that replaces the otherwise-missing protein packing interactions (Fig. 2D and fig. S8B). Homologs to NUTM have been identified in *Pichia angusta* (*Ogataea polymorpha*) and *Dekkera bruxellensis* (*Brettanomyces bruxellensis*) (*48*), and their AlphaFold3 (*59*) predictions show a similar four-helix architecture, with analogous changes to their subunits ND4L, ND6, and NDUFA1 to presumably accommodate the NUTM subunit accordingly (fig. S8C).

### The NUQM bridge is specific to the closed state

Like many supernumerary subunits (*24*, *27*), NUQM appears to have a structure-stabilizing role in CI. Unusually, however, it bridges the hydrophilic and membrane arms and appears specific to the closed state.

**Fig. 3.**
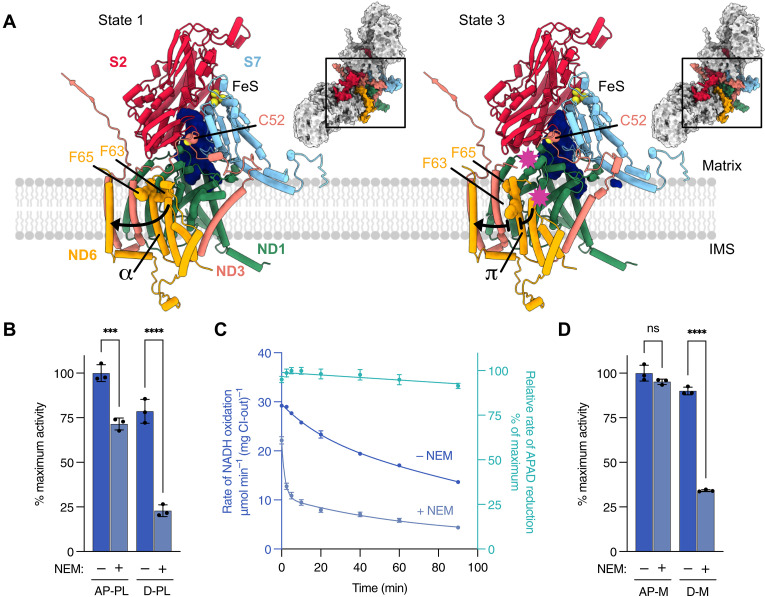
Q_10_-binding site loop conformations and exposure of C52^ND3^ to thiol-reactive NEM. (**A**) Organization of the Q_10_-binding site (dark blue surface) of the closed (state 1) and open (state 3) enzymes. The closed state shows ordered loops, a buried C52^ND3^, and α-helical TMH3^ND6^ that allows a continuous chain of protonatable groups to form along the membrane arm (black arrow). The open state includes disordering (between the pink stars) of the TMH1–2^ND3^ loop, resulting in the exposure of C52^ND3^, and of the TMH3–4^ND6^ loop. In addition, TMH3^ND6^ adopts a π conformation [residues Phe63^ND6^ (F63) and Phe65^ND6^ (F65) are shown rotated around the helical axis] that disrupts the chain (interrupted black arrow). IMS, intermembrane space. (**B**) NADH:O_2_ rates for as-prepared (AP) and deactivated (D) *Pp*-CI–containing PLs. Error bars indicate SD; *n* = 3. Significance test: one-way ANOVA. For deactivation, the *Pp*-CI-PLs were incubated at 37°C for 20 min. ***P = 0.0004; ****P < 0.0001. (**C**) Rates of the NADH:O_2_ and NADH:APAD^+^ reactions over time for *Pp*-CI-PLs incubated at 37°C for deactivation. Error bars indicate SD; *n* = 3. (**D**) dNADH:DQ rates for as-prepared and deactivated (37°C for 20 min) *Pp* mitochondrial membranes (M). Error bars indicate SD; *n* = 3. Significance test: one-way ANOVA. See Materials and Methods for experimental details of the assays used. ns, not significant; ****P < 0.0001.

Mammalian CI is known to adopt two biochemically defined resting states, the “active” and “deactive” resting states. The former is ready to begin catalyzing without delay and is insensitive to thiol-reactive reagents. The latter, which is trapped by thiol-reactive reagents that target a cysteine on the TMH1–2^ND3^ loop (C52^ND3^ for *Pp*-CI), requires NADH and Q_10_ to undergo reactivating turnovers that return it to the catalytic cycle (*40*, *60*, *61*). Extensive structural and functional characterization has defined the active resting state as the structurally closed state observed in cryo-EM (with a smaller apparent interdomain angle) and deactive resting states as the open states observed in cryo-EM (with larger apparent interdomain angles) (*39*, *62*–*66*). A set of local conformational signatures further distinguishes the closed and open states of the mammalian complex and is observed to a greater or lesser extent in the resting states of the complexes from other species (*18*). The NUQM-bound closed state 1 (and 2) of *Pp*-CI here includes all the local hallmarks of the closed active-state mammalian enzyme, including ordered Q_10_-binding loops that seal the Q_10_-binding channel from the matrix and a fully α-helical TMH3^ND6^ (without a π bulge) (Fig. 3A, fig. S9, and table S6) (*39*, *64*, *67*). The TMH1–2^ND3^ loop is well ordered and occludes C52^ND3^ from thiol-labeling agents. Therefore, we now refer to state 1 as the closed state of *Pp*-CI. In contrast, the NUQM-free state 3 (and 4) shows many local signatures of the mammalian open (deactive) state including the TMH3^ND6^ π bulge, Y156^ND1^ oriented out of the central membrane axis (fig. S9 and table S6), and a disordered TMH1–2^ND3^ loop (Fig. 3A) that makes C52^ND3^ susceptible to thiol labeling. The TMH3–4^ND6^ loop is disordered also, which is common in open states. Therefore, we now refer to state 3 as the open state of *Pp*-CI. In the open state of *Pp*-CI, the Q_10_ site loops in the NDUFS2, NDUFS7, and ND1 subunits are ordered and in their closed-like conformations, and as a result, the Q_10_ site stays closed to the matrix even in the globally open state. Similar behavior has been observed in the Open1 state of mammalian CI, as well as in the open states of other species (see below) (*18*, *39*, *50*, *56*, *68*). In summary, the results show that *Pp*-CI exhibits closed and open resting states that resemble the closed and open (active and deactive) resting states of the mammalian complex, respectively, and that NUQM binds to the closed state specifically. The presence of NUQM therefore correlates directly with the conformation of the enzyme’s resting state, which suggested that it stabilizes the closed conformation and its interaction with the complex is stabilized by it.

### Biochemical characterization of *Pp*-CI resting states

To evaluate the functional differences between the closed and open states of *Pp*-CI and to test whether they exhibit the same biochemical characteristics as the closed-active and open-deactive resting states of the mammalian complex, we reconstituted *Pp*-CI into proteoliposomes (PLs). On the basis of corresponding work on the mammalian complex, PLs enable robust functional assays and enhance enzyme stability at the elevated temperatures required for deactivation (*38*, *39*, *69*, *70*). First, we used the thiol-reactive agent NEM (*N*-ethylmaleimide) to evaluate the as-prepared *Pp*-CI in PLs (AP-PLs): 71.5 ± 3.6% of *Pp*-CI was insensitive to NEM and was therefore in the active resting state (Fig. 3B). Both the NEM-sensitive and NEM-insensitive activities were confirmed to be sensitive to piericidin A, a canonical CI inhibitor. In comparison, 68.1% of the (flavoprotein-containing) particles in the cryo-EM analysis were in the NUQM-bound closed state in which the target of NEM, C52^ND3^, is occluded on the well-ordered TMH1–2^ND3^ loop (Fig. 3A and fig. S3). The comparison supports the assignment of the cryo-EM NUQM-bound closed state to the biochemically defined active resting state. To confirm that the results were not affected by reconstitution of the enzyme into NDs versus PLs, we then performed single-particle cryo-EM on the *Pp*-CI-PLs using a 200-kV microscope (table S7). Even with the modest resolution this approach afforded, it was possible to separate out particles containing NUQM and/or the flavoprotein subcomplex (fig. S10). In excellent agreement with the biochemical data, 77% of the outward-facing, flavoprotein-bound particles contain the NUQM subunit also, supporting our proposed assignment. Last, to explore the purposeful deactivation of *Pp*-CI, we performed a heat treatment of the *Pp*-CI-PLs (37°C, 20 min) that would cause the bovine enzyme to become fully open and NEM sensitive/deactive (*38*, *39*, *63*). However, we did not observe complete deactivation of *Pp*-CI (~23% of it remained NEM insensitive), and there was also an unrecoverable partial loss of activity following the 20-min thermal deactivation procedure. Subsequently, a time course experiment (Fig. 3C) revealed a sharp but incomplete increase in NEM sensitivity within the first 10 min of heat treatment. Although later data are confounded by the general loss of NADH:O_2_ activity over time, which is not mirrored in the consistent rates of the flavin-localized NADH:APAD^+^ reaction, the proportion of NEM-sensitive enzyme does not increase further. Therefore, our biochemical analyses suggest that three types of isolated *Pp*-CI are present: an NUQM-bound closed/active enzyme resistant to deactivation (in which the energy barriers for opening are higher than those in bovine CI), an NUQM-bound closed/active enzyme susceptible to deactivation (in which the energy barriers for opening are comparable to those in bovine CI), and an NUQM-free open/deactive enzyme. Whether deactivation of the susceptible NUQM-bound closed/active enzyme causes the loss of NUQM from the nascent open enzyme is currently unknown. We speculate that the different behavior of the two closed states reflects subtle differences that have not been identified in our structural work, such as in the interface between NUQM and the rest of the complex, that may become more pronounced under deactivation conditions.

### Increased stability of the closed state in native membranes

**Fig. 4.**
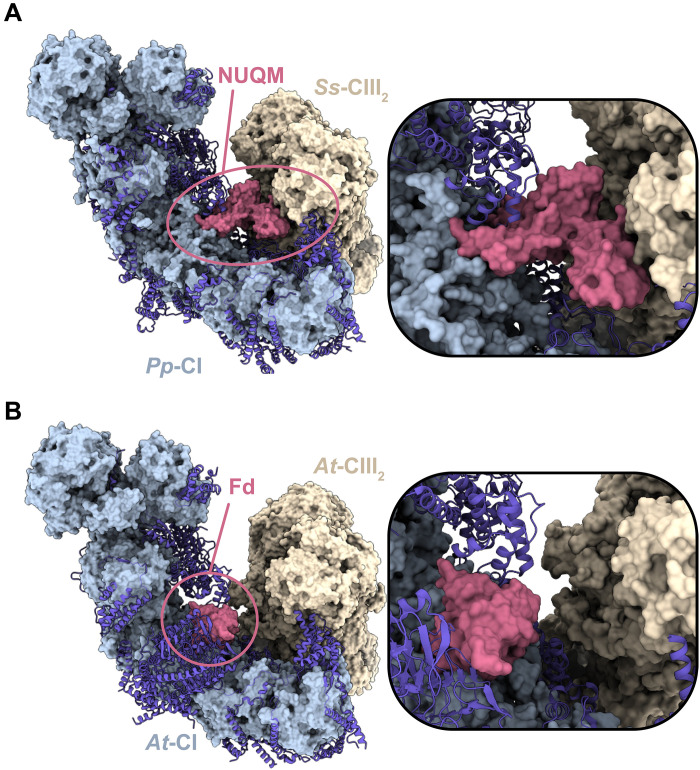
Proposed role of NUQM in supercomplex interactions in *Pp*-CI. (**A**) *Pp*-CI was first superpositioned onto the *Sus scrofa* (*Ss*) CI in the mammalian CI-CIII_2_ supercomplex (8UGH) before the removal of *Ss*-CI to observe the predicted interactions at the CI-CIII_2_ interface (inset). (**B**) CI-CIII_2_ interaction in the *A. thaliana* (*At*) supercomplex (8BQ5), where *At*-CI contains a ferredoxin (Fd) subunit that bridges its two major domains (inset). CI core subunits are shown in solid light blue, with supernumerary subunits in blue. NUQM and Fd are colored in the red surface, and CIII_2_ is shown in solid beige.

Recognizing that NUQM (like the flavoprotein domain) may become unstable or lost from *Pp*-CI during its extraction from the membrane or purification, we assessed the NEM sensitivity in native mitochondrial membrane preparations. In membranes, *Pp*-CI is ~95% insensitive to NEM following the standard NEM labeling procedure, indicating that it is essentially all in the active-closed resting state (Fig. 3D), and by comparison to the cryo-EM–defined states, we therefore assume that NUQM is stoichiometrically bound. As was true for the isolated enzyme, membrane activities were confirmed to be sensitive to piericidin A. The NEM-sensitive proportion increases markedly following the deactivation procedure, indicating that it is possible to open the membrane-bound complex, with the damaging effect of heat treatment substantially lower in the native membranes than in PLs. Therefore, it appears that as for the isolated enzyme in PLs, there are two classes of closed/active enzymes: one that is resistant to deactivation and one that is susceptible. As it has been shown biochemically and structurally that the complexes of the mitochondrial respiratory chain are predominantly found in higher-order supercomplex assemblies in native membranes (*65*, *71*, *72*), supercomplex associations may distinguish the two classes, and it is possible that removing *Pp*-CI from the membrane destabilizes or dislodges NUQM because it isolates CI from its supercomplex partners. While no high-resolution fungal supercomplex structures are available, lower-resolution investigations have suggested that similar architectures are adopted in fungi, mammals, and plants (*72*), with comparable high-resolution supercomplex structures available from plants (*15*, *73*), mammals (*65*, *66*, *74*, *75*), and now protists (*57*, *76*, *77*). In particular, CI and CIII_2_ are typically observed in a conserved configuration, contacting one another in the region where NUQM is bound in *Pp*-CI. Combining the structures of *Pp*-CI and the CI-CIII_2_ mammalian supercomplex in silico shows NUQM comfortably approaching CIII_2_, suggesting that it interacts with both CI domains and CIII_2_ within the supercomplex assembly (Fig. 4A). In comparison, the supercomplex of *Arabidopsis thaliana* (*15*) contains a ferredoxin protein in a similar domain-bridging location, but it does not make CIII_2_ contacts (Fig. 4B). Variation in the CI-CIII_2_ interface (and the stabilization of NUQM and the closed state) may thus explain the varying susceptibility of the NUQM-bound enzyme to deactivation, and breaking the CI-CIII_2_ interaction during purification may explain the different states of association of NUQM with *Pp*-CI observed in the isolated enzyme.

## DISCUSSION

Our cryo-EM analyses of isolated *Pp*-CI have revealed a unique supernumerary subunit, NUQM, that correlates to (and may control) the conformation of the enzyme’s resting state. When NUQM is bound, the enzyme adopts the canonical closed resting state that has been observed in many structures of CI from both prokaryotes and eukaryotes (*18*), whereas when NUQM is absent, the enzyme adopts a more “open” resting state with an increased apparent angle between the hydrophilic and membrane domains. Unlike the closed state, open states vary between species, with some differing from the closed state more than others and exhibiting a greater number of altered structural elements (*18*). The open state of *Pp*-CI observed here is an intermediate-level open state, with two elements in the membrane domain (TMH3^ND6^ in its π-bulge conformation and Tyr156^ND1^ flipped out of the E-channel) and the region of disorder observed in THM1–2^ND3^ (that facilitates NEM labeling) in open-type conformations but retaining the intact Tyr141^ND1^ trigonal junction (with Cys52^ND3^ and His107^NDUFS2^) and closed-like loops conformations around the Q_10_-binding site. The pattern of closed and open features, which switches characteristics at TMH4^ND1^, thus resembles that observed in the “twisted” state of CI from *Drosophila melanogaster* (*68*). Last, as also observed in twisted *D. melanogaster* and the Open1 state observed recently in mammalian CI (*39*), the Q_10_-binding site of open-state *Pp*-CI remains sealed to the matrix. We note that the terms closed and open have been used to refer both to the interdomain angle and to the status of the ubiquinone-binding site; the lack of correlation between them underlines the need to define and use the closed/open terminology carefully.

Direct correlation of structural data with functional data showed that only the open state of isolated *Pp*-CI is sensitive to NEM treatment, consistent with a disordered section in the TMH1–2^ND3^ loop that carries C52^ND3^. In this way, the behavior of the *Pp*-CI closed and open states matches that of the biochemically defined “active” (closed) and “deactive” (open) resting states of mammalian CI (*38*–*40*). These two states regulate the behavior of the mammalian enzyme under IR conditions in tissues and, therefore, its contribution to oxidative stress and IR injury: When O_2_ returns to the ischemic tissue and the proton-motive force is high, the closed, turnover-ready state is able to catalyze RET with an associated high rate of reactive oxygen species generation by the reduced flavin. However, the open resting state cannot catalyze RET (*38*, *39*), and therefore, the transition of the enzyme into this state during the ischemic phase is protective against IR injury during reperfusion (*9*, *31*, *40*). Our observation of a population of *Pp*-CI in the closed resting state is consistent with its capacity to perform RET (*38*), like the mammalian enzyme and CI from *P. denitrificans* (*78*, *79*). The presence of the closed state in *Pp* membranes further suggests that RET is possible in *Pp* cells whenever the bioenergetic environment is poised to support it but, similarly to that in mammalian mitochondria, at a cost of increased oxidative stress. In contrast, the enzyme from *Y. lipolytica* adopts only an open resting state, and attempts to induce it to catalyze RET have not been successful (*12*, *38*). The ability of CI to catalyze in reverse thus appears directly linked to whether it adopts a canonical closed resting state. Although we previously suggested that, alternatively, it may be linked to the presence of a hydroxylated arginine in NDUFS7 (*38*), further inspection of mass spectrometry data on *Pp*-CI (*48*) has showed, consistent with our structural data, that the Arg residue is not modified. Therefore, the presence of the hydroxylated arginine is not a determinant for RET, a result that is also consistent with the unmodified cryo-EM density of this residue in *P. denitrificans* (*58*).

Although no subunits homologous to *Pp*-CI NUQM have been found in CI before, different subunits that bridge the hydrophilic arm to the membrane arm have been observed in different species (fig. S11). In mammalian CI, the bridge is formed by NDUFA5 on the hydrophilic arm and NDUFA10 on the membrane arm. Unlike in isolated *Pp*-CI, the two bridging mammalian subunits are always present; their interface changes when the enzyme opens, and the relative disposition of the two subunits constitutes a simple marker for the state (*64*). The effect of NDUFA5 and NDUFA10 is thus more subtle than that of NUQM in *Pp*-CI: By always remaining bound, they stabilize the closed, ready-to-catalyze state by raising energy barriers to slow down the otherwise prompt opening (deactivation) transition, where the deactive state, which requires reactivation to catalyze in any direction, appears to have evolved as a regulatory mechanism in mammalian CI to protect against CI-mediated oxidative damage under challenging physiological conditions. It is not yet known whether NUQM binding is used in vivo to regulate the status of *Pp*-CI or whether the loss of the subunit upon opening occurs only in the isolated complex. In CI from the thermophilic fungus *Chaetomium thermophilum*, which also adopts closed and open states, an extension of NDUFA5 forms the bridge (*50*). In CI from plants and algae, a ferredoxin subunit bridges the domains, but in most cases, the canonical closed state has not yet been observed (*15*, *73*, *76*). The exception is the closed state of the ferredoxin-containing CI from the algae *Euglena gracilis* (*77*). Here, it was reported that the NEM sensitivity increased upon thermal deactivation. However, decreased rates were observed regardless of the NEM treatment; moreover, *E. gracilis* lacks the Cys^ND3^ residue, thereby precluding NEM sensitivity being used to properly differentiate the states, and the Q_10_-binding site loops did not change conformation during “deactivation.” Subunits specific to *E. gracilis*, such as the TMH-containing NDUEG6, may act to tether the hydrophilic arm to the membrane to restrict its motion and provide additional stabilization for the closed state (fig. S11D). A similar but reversible stabilizing effect was suggested in the mammalian enzyme with NDUFA9 acting as a membrane anchor for the hydrophilic domain and counterbalancing the regulatory opening transition (*39*). In *Pp*-CI, we see the NUTM subunit sitting underneath NDUFA9 (Fig. 2D), perhaps promoting its membrane anchor function. CI from *Tetrahymena thermophila* is another example of a closed-only enzyme (with no NEM sensitivity reported) (*57*). In this case, the extreme congestion of supernumerary subunits appears to lock the enzyme into the closed state (fig. S11E), presenting an obvious challenge to mechanistic proposals that necessitate rapid opening and closing (*11*, *13*). Other proposals have described catalysis within exclusively closed states, and there are so far no examples of structures locked specifically in open states, consistent with the catalytic relevance of the closed configuration.

More stably closed enzyme species may have accrued additional supernumerary subunits, such as bridging subunits, to tether, counterbalance, or restrict enzyme opening. However, the theme of supernumerary subunits evolving to stabilize the closed resting state is not the whole story (fig. S11G). For *E. gracilis* and *T. thermophila*, their CI configurations are extensively decorated/bridged with supernumerary subunits in supercomplexes, and their structures and biochemistry suggest that they do not have a state beyond the closed/active state. The considerable supernumerary subunits here facilitate discoid and tubular mitochondrial crista morphologies, which may provide a bioenergetic advantage in organisms less concerned with IR injury. In contrast, the CI from *Y. lipolytica* contains a similar number of supernumerary subunits to *Pp*-CI but rests exclusively in an open state; it is currently the only structure of eukaryotic CI with no domain-bridging supernumerary subunits in its catalog (*12*). Last, of the four known bacterial CI structures (*13*, *21*, *58*, *80*), none contain bridging supernumerary subunits, but two rest exclusively in the closed state (*P. denitrificans* and *Mycobacterium smegmatis*) and two rest in open states (*E. coli* and *Thermus thermophilus*). Closed states have been observed for *E. coli* CI but only after treatment with substrates. In *P. denitrificans*, a supernumerary subunit bound on the hydrophilic domain may stabilize the Q_10_-binding site loops (fig. S11F), and the corner between the domains is packed by the N-terminus of NDUFS2 (*23*, *58*). In *M. smegmatis*, a homolog of NDUFA9 may provide a counterbalance to the movement of the hydrophilic domain (*80*). In both these bacterial, closed-only enzymes, core subunit extensions cross between the domains and may also lend stability to their relative disposition. Therefore, although specific examples demonstrate their roles in individual cases, evolution has adopted a variety of strategies and supernumerary subunits that are neither sufficient or essential for stabilizing CI in the closed resting state.

To our knowledge, the P25L^ND6^ variant in mice is the first example of a targeted genetic change with a specific effect on the balance between the closed and open states in mammalian CI, in this case shifting the enzyme from a predominantly closed/active to an exclusively open/deactive resting state (*31*). The consequent functional changes provided notable insights into the physiological relevance of the deactive state: The P25L^ND6^ enzyme is able to catalyze forward normally but is unable to catalyze in reverse, lending protection to the tissue during reperfusion. Here, we have identified a binary correlation between the closed/open state of isolated *Pp*-CI and the presence/absence of the NUQM bridge subunit. Thus, in mitochondrial membranes where *Pp*-CI is insensitive to NEM and so in the closed state, we assume (by comparison to the cryo-EM–defined states) that NUQM is stoichiometrically bound, stabilizing the closed resting state and likely integration of CI into the respirasome. *Pp*-CI is therefore locked in a turnover-ready state, capable of reversible catalysis. Whether the presence/absence of the NUQM subunit is used as a tool to regulate *Pp*-CI in vivo is unknown. However, future investigations in which it is overexpressed, supplemented exogenously, or deleted from the *Pp* genome will enable direct evaluation of its molecular role in setting the state of CI, as well as the effects of locking CI in the closed state (or promoting its opening transition) on the organization and assembly of the respiratory complexes, the membrane topology, and mitochondrial homeostasis.

## MATERIALS AND METHODS

Unless otherwise stated, buffer pH values were corrected at room temperature, and purification and preparation protocols were carried out at 4°C.

### Preparation of *Pp*-CI

The *Pp* strain X-33 was batch grown in a fermenter and stored at −70°C (*47*). To isolate the membranes (*38*, *47*), cells were thawed; resuspended at 500 g liter^−1^ in 20 mM 3-morpholinopropane-1-sulfonic acid (Mops; pH 7.2), 400 mM sorbitol, 5 mM EDTA, 5 mM benzamidine, 2% bovine serum albumin, 5 mM ε-aminocaproic acid, and 800 μM phenylmethylsulfonyl fluoride; and passed twice through a Dyno-Mill bead mill. Following centrifugation to remove debris (6000*g*, 15 min), the membranes were collected by centrifugation (235,000*g*, 1 hour) and washed twice by homogenizing them into 20 mM Mops (pH 7.2), 400 mM sorbitol, 5 mM ε-aminocaproic acid, and 5 mM benzamidine and recollecting them (235,000*g*, 35 min). The final membranes were homogenized in 20 mM NaH_2_PO_4_ (pH 7.45) and 50 mM NaCl and frozen for storage.

The protocol for purifying *Pp*-CI in LMNG was adapted from the DDM-based protocol used previously (*38*). Defrosted membranes were diluted to 15 mg-protein ml^−1^, and a tablet of cOmplete EDTA-free protease inhibitor cocktail was added per 25 ml. LMNG was added dropwise to a final concentration of 2%, and the solubilization mixture was stirred for 30 min and then centrifuged (210,000*g*, 45 min). NaCl and imidazole were added to the supernatant to 400 mM and 20 mM, respectively, and then the sample was filtered (0.22 μm) and loaded onto a 10-ml HisTrap Ni-affinity column (Cytiva), pre-equilibrated with 20 mM NaH_2_PO_4_ (pH 7.2), 400 mM NaCl, 42 mM imidazole, 0.02% asolectin (Avanti Polar Lipids), 0.02% 3-[3-cholamidopropyl dimethylammonio]-1-propanesulfonate (CHAPS; Merck), and 0.15% LMNG. The column was washed until the 280- and 420-nm absorbances baselined and then eluted in the same buffer containing 140 mM (rather than 42 mM) imidazole. *Pp*-CI–containing fractions were pooled, concentrated using a 100-kDa molecular weight cutoff (MWCO) concentrator, and eluted from a Superose 6 Increase 10/300 GL column (Cytiva) in 20 mM Mops (pH 7.4), 150 mM NaCl, and 0.0375% LMNG. The peak fractions containing *Pp*-CI were pooled, concentrated, and frozen for storage.

### Preparation of AOX

AOX from *Trypanosoma brucei* was expressed in *E. coli* membranes and purified by adapting the Twin-Strep tag affinity chromatography protocol described previously (*81*). Briefly, membranes (6 mg-protein ml^−1^) were resuspended in 25 mM tris-HCl (pH 7.5), 200 mM MgSO_4_, and 10% glycerol. DDM was added dropwise to a final concentration of 1%, and then the solubilization mixture was stirred for 30 min and centrifuged (220,000*g*, 45 min). The supernatant was filtered (0.22 μm) and loaded onto a 5-ml Strep-Tactin Superflow affinity column (IBA Life Sciences) pre-equilibrated with 20 mM tris-HCl (pH 7.5), 160 mM NaCl, 5 mM MgSO_4_, 10% glycerol, and 0.042% DDM. The column was washed and then eluted with the same buffer and 2.5 mM desthiobiotin. Pooled AOX-containing fractions were concentrated (10-kDa MWCO), and then desthiobiotin was removed using a PD10 desalting column (Cytiva). AOX was concentrated and frozen for storage.

### Preparation of cMSP26

Five hundred milliliters of LB broth containing kanamycin (50 μg ml^−1^) and chloramphenicol (50 μg ml^−1^) was inoculated with a 5-ml pre-culture of *E. coli* BL21-CodonPlus RIL (DE3) containing the cMSP26 plasmid (*82*). The cells were grown aerobically (37°C, 180 rpm shaking) until cMSP26 expression was induced by 1 mM isopropyl-β-d-1-thiogalactopyranoside at an optical density at 600 nm of ~0.6. The cells were grown for a further 16 to 18 hours (25°C, 180 rpm), harvested by centrifugation (17,000*g*, 10 min), and then resuspended in 50 mM tris-HCl (pH 8), 100 mM NaCl, 5 mM MgCl_2_, 1% Triton X-100, cOmplete EDTA-free protease inhibitor cocktail, 0.002% phenylmethylsulfonyl fluoride, lysozyme (l mg ml^−1^), and a few crystals of deoxyribonuclease I. The suspension was sonicated using a Q700 probe sonicator (QSonica) equipped with a 1.6-mm microtip (60% amplitude, 2.5 min, 20 s on/20 s off). The lysate was centrifuged (10,000*g* for 15 min and then 250,000*g* for 1 hour), and then the supernatant was heated to 70°C for 15 min to denature the proteins and centrifuged again (10,000*g*, 15 min at 25°C). The supernatant was filtered (0.22 μm) and loaded onto a 5-ml HisTrap Ni-affinity column (Cytiva), pre-equilibrated with 20 mM tris-HCl (pH 8) and 300 mM NaCl. To promote cMSP26 binding, the sample was cycled through the column for 1 hour at 2 ml min^−1^, and then the column was washed until the 280-nm absorbance baselined. Proteins were eluted by increasing the imidazole concentration stepwise through increasing the proportion of a second buffer (the same composition and 500 mM imidazole). cMSP26-containing fractions eluting at 60 mM were pooled, concentrated (50-kDa MWCO), and frozen for storage.

### Preparation of *Pp*-CI-NDs and *Pp*-CI-PLs

A mixture of synthetic lipids comprising 1,2-dioleoyl-*sn*-glycero-3phosphocholine (DOPC), 1,2-dioleoyl-*sn*-glycero-3-phosphoethanol-amine (DOPE), and 18:1 cardiolipin (CDL) (Avanti Polar Lipids) at a mass ratio of 8:1:1 (DOPC:DOPE:CDL) was dissolved in chloroform at 25 mg ml^−1^. Q_10_, also dissolved in chloroform, was added at 40 nmol (mg lipid)^−1^ (*Pp*-CI-NDs, 5 mg of lipids) or 10 nmol (mg lipid)^−1^ (*Pp*-CI-PLs, 10 mg of lipids), and chloroform was then removed, first under a N_2_ stream and then in a desiccator under vacuum for >1 hour.

For *Pp*-CI-NDs, the dried lipid mixture was resuspended in 5 ml of resuspension buffer [10 mM Mops (pH 7.5), 50 mM KCl, and 12 mM sodium cholate], sonicated in an ultrasonic bath for 10 min, and stored at −20°C. The protocol for reconstitution was adapted from a published protocol (*49*). Five hundred microliters of suspension was centrifuged to remove insoluble material (7000*g*, 10 min), and then cMSP26 and *Pp*-CI were added at a molar ratio of 1200 lipids:10 cMSP26:1 *Pp*-CI and mixed gently. The sample was diluted twofold by the addition of further buffer, incubated on ice for 20 min, and passed through a PD10 desalting column (Cytiva) in 10 mM Mops (pH 7.5) and 50 mM KCl. The *Pp*-CI-NDs were concentrated to ~100 μl (100-kDa MWCO), filtered (0.22 μm), and eluted from a Superose 6 increase 5/150 column (Cytiva) in the same buffer. *Pp*-CI-ND–containing peak fractions were used directly for cryo-EM grid preparation.

*Pp*-CI-PLs were prepared using a protocol adapted from that used previously (*39*). The dried lipid-Q_10_ mixture was hydrated in 1 ml of resuspension buffer for 30 min with regular vortexing and then sonicated on ice using a Q700 probe sonicator equipped with a 1.6-mm microtip (60% amplitude, 2.5 min, 15 s on/30 s off). Five hundred microliters of the resulting liposomes (10 mg-lipid ml^−1^) was incubated with 0.5% (final concentration) sodium cholate on ice for 10 min, then *Pp*-CI was added at a mass ratio of 50:1 lipids:CI, and the mixture was incubated again for 10 min. The detergent was then removed by passing the sample through a PD10 desalting column (Cytiva) in 10 mM Mops (pH 7.5) and 50 mM KCl. The resulting sample was centrifuged (150,000*g*, 1 hour) and resuspended in 100 μl of the same buffer for use.

### CI activity assays

All catalytic assays were performed at 32°C in 96-well microtiter plates using a Molecular Devices SpectraMax ABS Plus microplate reader. Rates of NADH oxidation were determined at 340 to 380 nm (ε_340–380_ = 4.81 mM^−1^ cm^−1^), unless otherwise stated. Assays on isolated CI were performed in 20 mM tris-HCl (pH 7.5 at 32°C), and assays on membrane systems (including *Pp*-CI-NDs and PLs) were in 10 mM Mops (pH 7.5) and 50 mM KCl. Assays on native *Pp* membranes used deamino-NADH (dNADH) in place of NADH to avoid contributions from other NADH-linked enzymes (*47*, *83*). Piericidin A (500 nM) was added to inhibit *Pp*-CI catalysis. NADH:APAD^+^ (3-acetylpyridine adenine dinucleotide) oxidoreduction rates were measured with 100 μM NADH and 500 μM APAD^+^. Piericidin A (500 nM) was added, and 0.2% DDM was added for measurements in detergent. The rates of NADH:APAD^+^ oxidoreduction were compared with and without alamethicin (15 μg ml^−1^), a pore-forming antibiotic, to determine the inward/outward distribution of CI in PLs (*38*, *81*). NADH:DQ oxidoreduction rates were measured with 200 μM NADH and 200 or 400 μM DQ. Native membranes (10 μg ml^−1^) and *Pp*-CI (0.5 μg ml^−1^) were assayed in the presence of 0.15% (w/v) asolectin and 0.15% CHAPS to aid the solubility of DQ (*84*). Asolectin and CHAPS were not required for measurements of *Pp*-CI-NDs (0.5 μg ml^−1^). Rates of Q_10_-mediated NADH:O_2_ oxidoreduction in *Pp*-CI-NDs and *Pp*-CI-PLs were measured with 200 μM NADH using *T. brucei brucei* AOX (5 μg ml^−1^) at 0.5 μg ml^−1^ [0.5 μg ml^−1^ CI-out for PLs]. For deactivation trials (*39*), *Pp*-CI-PLs (200 μg ml^−1^ CI-out) and native membranes (2 mg-protein ml^−1^) were incubated at 37°C for 20 min or as stated. To determine the sensitivity to NEM, samples were incubated on ice for 30 min with 1 mM NEM (added from a stock in which 400 mM NEM in dimethyl sulfoxide was diluted to 100 mM in assay buffer) before determining the CI activity (*38*).

### Cryo-EM grid preparation, screening, and imaging

For the *Pp*-CI-ND dataset, carbon copper grids (0.6/1 Quantifoil) were washed with chloroform (30 s) and then ethanol (30 s) and glow discharged twice at 20 mA (90 s) before sample application (2.5 μl of 0.7 mg ml^−1^ protein). Grids were frozen in an FEI Vitrobot Mark IV (Thermo Fisher Scientific), set to 95% humidity (4°C) with a blot force of −10 for 2 s before plunge-freezing in liquid ethane (−186°C). For the *Pp*-CI-PL dataset, gold carbon grids (1.2/1.3 Quantifoil) were washed with chloroform (30 s) and then ethanol (30 s) and dried in air. Grids were glow discharged for 20 mA, 90 s, and 0.39 mbar before the application of a graphene oxide solution (0.4 mg ml^–1^, Sigma-Aldrich) that was blotted and washed three times with water and dried at 100°C for 30 min. Before sample addition (2 μl per grid, 100 μg ml^−1^ CI-out determined via the rate of NADH:APAD^+^ oxidoreduction; see above), the graphene oxide solution–coated grids were glow discharged (10 mA, 15 s, 0.39 mbar). Grids were frozen in an FEI Vitrobot Mark IV, set to 90% humidity (22°C) with a wait time of 30 s, a blot force of 0, and a blot time of 2 s before plunge-freezing in liquid ethane (−186°C). Grids were screened at the cryo-EM facility at the Department of Biochemistry, University of Cambridge, using a Talos Arctica 200-kV microscope (Thermo Fisher Scientific).

### Cryo-EM data collection and processing of the *Pp*-CI-ND dataset

Data were collected overnight on a single grid using a Titan Krios microscope at the Department of Biochemistry, University of Cambridge. The microscope was operated in counting mode at 300 kV, with a Falcon 4i detector mounted with 100- and 50-μm objective and C2 apertures, respectively. The pixel size was 0.929 Å/pixel (130,000× nominal magnification), and the defocus range was −0.6 to −1.8 μm in 0.2 increments. Images were acquired with one shot per hole in aberration-free image shift mode, with 1-s delay after image shift. The energy filter was operated with a slit width of 10 eV, and a dose rate of 8.17 e-Å^−2^ s^−1^ was used, exposing the grid to a total dose of 47.8 e-Å^−2^ in 5.85 s in 40 fractions. A total of 7574 movies was collected and processed.

Cryo-EM data were processed mostly in RELION version 4.0 (*85*), except for specific tasks, performed in CryoSPARC version 3.3.2 (fig. S3) (*86*). Beam-induced motions of the micrographs were corrected using the RELION implementation (*87*) of MotionCor2 (*88*) with the gain-reference provided. The CTF of the motion-corrected micrographs was estimated using CTFFIND-4.1.0 (*89*). Initially, two-dimensional (2D) reference-based automated particle picking was performed using 2D class projections of bovine CI (EMD-14132) on a subset of 50 micrographs. The picked particles were then used for iterative Topaz training (*90*) and extracted with a box size of 540 pixels (rescaled to 64 pixels).

Picked particles were imported to CryoSPARC for 2D classification, ab initio reconstruction, and heterogeneous refinements. 2D classification was repeated to remove junk particles, and then selected particles were re-extracted with recentering in RELION at a box size of 540 pixels and used to generate an ab initio reference volume. Good particles identified from heterogeneous refinements were further subjected to repeated rounds of 3D classification using masks covering the flavoprotein (NDUFV1 and NDUFV2) and NUQM. RELION was then used for gold-standard 3D autorefinement before Bayesian polishing and CTF refinement (*87*, *91*), including beam tilt, trefoil, and per-particle defocus estimations. Autorefinement was then repeated before global and focused 3D classification without alignments (fig. S3): Each class was classified over four classes, and misclassified particles were reclassified on the basis of the presence of the flavoprotein or NUQM. The focus-subtract-classify-revert method was used for the final particle reclassification (*39*), focusing on differences at the membrane/hydrophilic domain interface. First, the particles were focus refined on the hydrophilic domain, and then they were subtracted with the proximal membrane domain mask and classified over four classes with a regularization parameter (*T*) value of 100. The output classes were reverted to their original states and 3D refined before being reclassified. The subsequent classes were 3D autorefined again before postprocessing to estimate the global resolutions [FSC (Fourier shell correlation) cutoff = 0.143]: state 1, 2.80 Å; state 2, 2.90 Å; state, 3.34 Å; state 4, 3.77 Å. Local resolutions were estimated using the auto–B-factors (state 1: −1.44, state 2: −9.83, state 3: −9.88; state 4: −28.69) calculated from the respective postprocess jobs and colored onto maps using UCSF ChimeraX version 1.9 (*92*).

Masks were generated using the molmap command in UCSF ChimeraX and models for *Y. lipolytica* CI [Protein Data Bank (PDB) ID: 6YJ4] and the resulting model from this dataset, outlined in more detail below. For global masks, a soft edge of 3 pixels was used, with focused masks using a value of 10 pixels. Other masks used were autogenerated in CryoSPARC.

### Cryo-EM data collection and processing of the *Pp*-CI-PL dataset

Data were collected overnight on a single grid using a Talos Arctica microscope at the Department of Biochemistry, University of Cambridge. The microscope was operated in linear mode at 200 kV, with a Falcon 3EC detector mounted with 100- and 50-μm objective and C2 apertures, respectively. The pixel size was 1.37 Å/pixel (73,000× nominal magnification), and the defocus range was −1.8 to −3.3 μm in 0.3 increments. The images were acquired with one shot per hole and a dose rate of 29.8 e-Å^−2^ s^−1^, exposing the grid to a total dose of 50.37 e-Å^−2^ in 1.69 s over 33 fractions. A total of 854 gain-corrected movies was collected and taken forward for processing.

Cryo-EM data were processed in RELION version 4.0 except for specific tasks, including 2D classification and heterogeneous refinement, carried out in CryoSPARC version 3.3.2 (fig. S10). Beam-induced motions of the micrographs were corrected using the RELION implementation of MotionCor2. The CTF of the motion-corrected micrographs was estimated using CTFFIND-4.1.0. Initially, Topaz was used to pick particles with a model trained on a previous bovine CI PL dataset (*39*). The picked particles were extracted (3.85 Å/pixel) and 2D classified in CryoSPARC. Good particles were then used to train a Topaz model before repeating the process. Particles were then re-extracted and recentered (1.37 Å/pixel) before 3D autorefinement, polishing, and CTF refinement in RELION. The particles were subjected to focused 3D classification without alignments using masks for either the flavoprotein or NUQM (fig. S10). Classes were then grouped and autorefined into four consensus maps before global sharpening: flavoprotein-bound, 4.7 Å; flavoprotein-free, 19.7 Å; NUQM-bound, 4.7 Å; NUQM-free, 8.2 Å (FSC cutoff = 0.143). To determine the inward- and outward-facing distribution of the classes, the particles were combined for each state and then heterogeneously refined using references of inward- and outward-facing bovine CI (*39*). Because of the low resolution of the output classes, performing focused classification on the flavoprotein and NUQM was not possible; therefore, we used the intersecting particles from the flavoprotein or NUQM classifications performed initially to obtain the final particle numbers (fig. S10).

Masks were generated using the state 1 model determined here (PDB ID: 9IHR) and the molmap command in UCSF ChimeraX. A soft edge of 5 pixels was used. Other masks used were autogenerated in CryoSPARC.

### Model building, refinement, and validation

ModelAngelo (*51*), implemented in RELION version 5.0, was used to generate an initial model using the globally postprocessed map of state 1 in RELION and sequences of *Pp*-CI obtained from Bridges *et al.* (*48*). For subunits that were not identified previously (NDUFC2/NEBM, NDUFB2/NIGM, and NUQM), sequences were obtained through BLASTp (*93*) searches of the sequences predicted from the raw CIF model output by ModelAngelo. ModelAngelo built fragmented chains at the peripheries of the complex because of lower resolution, and so a composite map was generated from locally refined maps (fig. S5) using the Combine Focus Map command in Phenix version 1.20-4487 (*94*) and the initial model from *M*odelAngelo as the reference. ModelAngelo was run again on the composite map with the recently identified sequences provided, and the output model file was carefully inspected in Coot version 0.9.8.92 (*95*) and manually refined and built where necessary. Phospholipids including phosphatidylcholine, phosphatidylethanolamine, and CDL were added de novo in Coot. Cofactors including FMN, FeS clusters, NADPH, 4′-phosphopantetheine + 3-hydroxytetradecanoate, K^+^, and Zn^2+^ were added also. All model refinements were performed in Phenix version 1.21-5207 (*94*)*.* Initially, hydrogens were added to the models using the ReadySet tool, and real-space refinement was performed against a composite map where present using the ligand CIF and EDITS restraint files for ligand-protein interactions. Subsequent refinements used the globally sharpened maps generated with RELION, and hydrogens were removed.

### Structure analysis and visualization

All models and maps were visualized in UCSF ChimeraX. Protein models were superpositioned using the MatchMaker command. The interior surfaces of the quinone-binding cavity and other relevant regions of the complex were predicted with the CASTp cavity searching tool (*96*) with a default 1.4-Å probe radius. Hydrogen bonds were identified using the hbonds command with default settings in UCSF ChimeraX. Figures were generated with Affinity Designer version 2. AlphaFold3 predictions were made using the web server (*59*). The MapQ plug-in was used to assess *Q*-scores (model atom-map correlation) (*97*) using UCSF Chimera version 1.13 (*98*).

### Statistical analysis

For biochemical assays, *n* = 3 and SD intervals are indicated. Significance was tested using a one-way analysis of variance (ANOVA) with Tukey’s multiple comparisons.

## References

[1] J. Hirst, Mitochondrial complex I. Annu. Rev. Biochem. 82, 551–575 (2013). 23527692 10.1146/annurev-biochem-070511-103700

[2] K. Parey, C. Wirth, J. Vonck, V. Zickermann, Respiratory complex I—Structure, mechanism and evolution. Curr. Opin. Struct. Biol. 63, 1–9 (2020). 32058886 10.1016/j.sbi.2020.01.004

[3] D. Nicholls, S. Ferguson, *Bioenergetics* (Elsevier, ed. 4, 2013); https://linkinghub.elsevier.com/retrieve/pii/C20100649029.

[4] A. H. V. Schapira, Human complex I defects in neurodegenerative diseases. Biochim. Biophys. Acta 1364, 261–270 (1998). 9593927 10.1016/s0005-2728(98)00032-2

[5] E. Fassone, S. Rahman, Complex I deficiency: Clinical features, biochemistry and molecular genetics. J. Med. Genet. 49, 578–590 (2012). 22972949 10.1136/jmedgenet-2012-101159

[6] R. J. Rodenburg, Mitochondrial complex I-linked disease. Biochim. Biophys. Acta 1857, 938–945 (2016). 26906428 10.1016/j.bbabio.2016.02.012

[7] K. Fiedorczuk, L. A. Sazanov, Mammalian mitochondrial complex I structure and disease-causing mutations. Trends Cell Biol. 28, 835–867 (2018). 30055843 10.1016/j.tcb.2018.06.006

[8] E. T. Chouchani, V. R. Pell, E. Gaude, D. Aksentijević, S. Y. Sundier, E. L. Robb, A. Logan, S. M. Nadtochiy, E. N. J. Ord, A. C. Smith, F. Eyassu, R. Shirley, C.-H. Hu, A. J. Dare, A. M. James, S. Rogatti, R. C. Hartley, S. Eaton, A. S. H. Costa, P. S. Brookes, S. M. Davidson, M. R. Duchen, K. Saeb-Parsy, M. J. Shattock, A. J. Robinson, L. M. Work, C. Frezza, T. Krieg, M. P. Murphy, Ischaemic accumulation of succinate controls reperfusion injury through mitochondrial ROS. Nature 515, 431–435 (2014). 25383517 10.1038/nature13909 PMC4255242

[9] S. Dröse, A. Stepanova, A. Galkin, Ischemic A/D transition of mitochondrial complex I and its role in ROS generation. Biochim. Biophys. Acta 1857, 946–957 (2016). 26777588 10.1016/j.bbabio.2015.12.013 PMC4893024

[10] D. N. Grba, J. Hirst, Mitochondrial complex I structure reveals ordered water molecules for catalysis and proton translocation. Nat. Struct. Mol. Biol. 27, 892–900 (2020). 32747785 10.1038/s41594-020-0473-x PMC7612091

[11] D. Kampjut, L. A. Sazanov, The coupling mechanism of mammalian respiratory complex I. Science 370, eabc4209 (2020). 32972993 10.1126/science.abc4209

[12] K. Parey, J. Lasham, D. J. Mills, A. Djurabekova, O. Haapanen, E. G. Yoga, H. Xie, W. Kühlbrandt, V. Sharma, J. Vonck, V. Zickermann, High-resolution structure and dynamics of mitochondrial complex I—Insights into the proton pumping mechanism. Sci. Adv. 7, 3221 (2021). 10.1126/sciadv.abj3221 PMC8589321 34767441

[13] V. Kravchuk, O. Petrova, D. Kampjut, A. Wojciechowska-Bason, Z. Breese, L. Sazanov, A universal coupling mechanism of respiratory complex I. Nature 609, 808–814 (2022). 36104567 10.1038/s41586-022-05199-7

[14] D. N. Grba, I. Chung, H. R. Bridges, A.-N. A. Agip, J. Hirst, Investigation of hydrated channels and proton pathways in a high-resolution cryo-EM structure of mammalian complex I. Sci. Adv. 9, eadi1359 (2023). 37531432 10.1126/sciadv.adi1359 PMC10396290

[15] N. Klusch, M. Dreimann, J. Senkler, N. Rugen, W. Kühlbrandt, H.-P. Braun, Cryo-EM structure of the respiratory I + III_2_ supercomplex from *Arabidopsis thaliana* at 2 Å resolution. Nat. Plants 9, 142–156 (2022). 36585502 10.1038/s41477-022-01308-6 PMC9873573

[16] A. S. Galkin, V. G. Grivennikova, A. D. Vinogradov, →H^+^/2e^–^ stoichiometry in NADH-quinone reductase reactions catalyzed by bovine heart submitochondrial particles. FEBS Lett. 451, 157–161 (1999). 10371157 10.1016/s0014-5793(99)00575-x

[17] A. J. Y. Jones, J. N. Blaza, F. Varghese, J. Hirst, Respiratory complex I in *Bos taurus* and *Paracoccus denitrificans* pumps four protons across the membrane for every NADH oxidized. J. Biol. Chem. 292, 4987–4995 (2017). 28174301 10.1074/jbc.M116.771899 PMC5377811

[18] I. Chung, D. N. Grba, J. J. Wright, J. Hirst, Making the leap from structure to mechanism: Are the open states of mammalian complex I identified by cryoEM resting states or catalytic intermediates? Curr. Opin. Struct. Biol. 77, 102447 (2022). 36087446 10.1016/j.sbi.2022.102447 PMC7614202

[19] H. Kim, P. Saura, M. C. Pöverlein, A. P. Gamiz-Hernandez, V. R. I. Kaila, Quinone catalysis modulates proton transfer reactions in the membrane domain of respiratory complex I. J. Am. Chem. Soc. 145, 17075–17086 (2023). 37490414 10.1021/jacs.3c03086 PMC10416309

[20] R. G. Efremov, R. Baradaran, L. A. Sazanov, The architecture of respiratory complex I. Nature 465, 441–445 (2010). 20505720 10.1038/nature09066

[21] R. Baradaran, J. M. Berrisford, G. S. Minhas, L. A. Sazanov, Crystal structure of the entire respiratory complex I. Nature 494, 443–448 (2013). 23417064 10.1038/nature11871 PMC3672946

[22] M. Sato, J. Torres-Bacete, P. K. Sinha, A. Matsuno-Yagi, T. Yagi, Essential regions in the membrane domain of bacterial complex I (NDH-1): The machinery for proton translocation. J. Bioenerg. Biomembr. 46, 279–287 (2014). 24973951 10.1007/s10863-014-9558-8

[23] O. D. Jarman, O. Biner, J. J. Wright, J. Hirst, *Paracoccus denitrificans*: A genetically tractable model system for studying respiratory complex I. Sci. Rep. 11, 10143 (2021). 33980947 10.1038/s41598-021-89575-9 PMC8115037

[24] J. Hirst, Why does mitochondrial complex I have so many subunits? Biochem. J. 437, e1–e3 (2011). 21711245 10.1042/BJ20110918

[25] D. A. Stroud, E. E. Surgenor, L. E. Formosa, B. Reljic, A. E. Frazier, M. G. Dibley, L. D. Osellame, T. Stait, T. H. Beilharz, D. R. Thorburn, A. Salim, M. T. Ryan, Accessory subunits are integral for assembly and function of human mitochondrial complex I. Nature 538, 123–126 (2016). 27626371 10.1038/nature19754

[26] Q. L. Dang, D. H. Phan, A. N. Johnson, M. Pasapuleti, H. A. Alkhaldi, F. Zhang, S. B. Vik, Analysis of human mutations in the supernumerary subunits of complex I. Life 10, 296 (2020). 33233646 10.3390/life10110296 PMC7699753

[27] A. Padavannil, M. G. Ayala-Hernandez, E. A. Castellanos-Silva, J. A. Letts, The mysterious multitude: Structural perspective on the accessory subunits of respiratory complex I. Front. Mol. Biosci. 8, 1–33 (2022). 10.3389/fmolb.2021.798353 PMC8762328 35047558

[28] M. A. Tocilescu, U. Fendel, K. Zwicker, S. Kerscher, U. Brandt, Exploring the ubiquinone binding cavity of respiratory complex I. J. Biol. Chem. 282, 29514–29520 (2007). 17681940 10.1074/jbc.M704519200

[29] I. H. Jain, L. Zazzeron, R. Goli, K. Alexa, S. Schatzman-Bone, H. Dhillon, O. Goldberger, J. Peng, O. Shalem, N. E. Sanjana, F. Zhang, W. Goessling, W. M. Zapol, V. K. Mootha, Hypoxia as a therapy for mitochondrial disease. Science 352, 54–61 (2016). 26917594 10.1126/science.aad9642 PMC4860742

[30] K. Parey, O. Haapanen, V. Sharma, H. Köfeler, T. Züllig, S. Prinz, K. Siegmund, I. Wittig, D. J. Mills, J. Vonck, W. Kühlbrandt, V. Zickermann, High-resolution cryo-EM structures of respiratory complex I: Mechanism, assembly, and disease. Sci. Adv. 5, eaax9484 (2019). 31844670 10.1126/sciadv.aax9484 PMC6905873

[31] Z. Yin, N. Burger, D. Kula-Alwar, D. Aksentijević, H. R. Bridges, H. A. Prag, D. N. Grba, C. Viscomi, A. M. James, A. Mottahedin, T. Krieg, M. P. Murphy, J. Hirst, Structural basis for a complex I mutation that blocks pathological ROS production. Nat. Commun. 12, 707 (2021). 33514727 10.1038/s41467-021-20942-w PMC7846746

[32] Z. Yin, A.-N. A. Agip, H. R. Bridges, J. Hirst, Structural insights into respiratory complex I deficiency and assembly from the mitochondrial disease-related *ndufs4*^−/−^ mouse. EMBO J. 43, 225–249 (2024). 38177503 10.1038/s44318-023-00001-4 PMC10897435

[33] S. Kerscher, S. Dröse, K. Zwicker, V. Zickermann, U. Brandt, *Yarrowia lipolytica*, a yeast genetic system to study mitochondrial complex I. Biochim. Biophys. Acta 1555, 83–91 (2002). 12206896 10.1016/s0005-2728(02)00259-1

[34] V. Zickermann, C. Wirth, H. Nasiri, K. Siegmund, H. Schwalbe, C. Hunte, U. Brandt, Mechanistic insight from the crystal structure of mitochondrial complex I. Science 347, 44–49 (2015). 25554780 10.1126/science.1259859

[35] U. Fendel, M. A. Tocilescu, S. Kerscher, U. Brandt, Exploring the inhibitor binding pocket of respiratory complex I. Biochim. Biophys. Acta 1777, 660–665 (2008). 18486594 10.1016/j.bbabio.2008.04.033

[36] J. Schiller, E. Laube, I. Wittig, W. Kühlbrandt, J. Vonck, V. Zickermann, Insights into complex I assembly: Function of NDUFAF1 and a link with cardiolipin remodeling. Sci. Adv. 8, eaad3855 (2022). 10.1126/sciadv.add3855 PMC9668296 36383672

[37] E. Maklashina, A. B. Kotlyar, G. Cecchini, Active/de-active transition of respiratory complex I in bacteria, fungi, and animals. Biochim. Biophys. Acta 1606, 95–103 (2003). 14507430 10.1016/s0005-2728(03)00087-2

[38] J. J. Wright, O. Biner, I. Chung, N. Burger, H. R. Bridges, J. Hirst, Reverse electron transfer by respiratory complex I catalyzed in a modular proteoliposome system. J. Am. Chem. Soc. 144, 6791–6801 (2022). 35380814 10.1021/jacs.2c00274 PMC9026280

[39] D. N. Grba, J. J. Wright, Z. Yin, W. Fisher, J. Hirst, Molecular mechanism of the ischemia-induced regulatory switch in mammalian complex I. Science 384, 1247–1253 (2024). 38870289 10.1126/science.ado2075

[40] A. B. Kotlyar, A. D. Vinogradov, Slow active/inactive transition of the mitochondrial NADH-ubiquinone reductase. Biochim. Biophys. Acta 1019, 151–158 (1990). 2119805 10.1016/0005-2728(90)90137-s

[41] E. T. Chouchani, C. Methner, S. M. Nadtochiy, A. Logan, V. R. Pell, S. Ding, A. M. James, H. M. Cochemé, J. Reinhold, K. S. Lilley, L. Partridge, I. M. Fearnley, A. J. Robinson, R. C. Hartley, R. A. J. Smith, T. Krieg, P. S. Brookes, M. P. Murphy, Cardioprotection by S-nitrosation of a cysteine switch on mitochondrial complex I. Nat. Med. 19, 753–759 (2013). 23708290 10.1038/nm.3212 PMC4019998

[42] M. P. Murphy, How mitochondria produce reactive oxygen species. Biochem. J. 417, 1–13 (2009). 19061483 10.1042/BJ20081386 PMC2605959

[43] E. T. Chouchani, V. R. Pell, A. M. James, L. M. Work, K. Saeb-Parsy, C. Frezza, T. Krieg, M. P. Murphy, A unifying mechanism for mitochondrial superoxide production during ischemia-reperfusion injury. Cell Metab. 23, 254–263 (2016). 26777689 10.1016/j.cmet.2015.12.009

[44] C. P. Kurtzman, Description of *Komagataella phaffii* sp. nov. and the transfer of *Pichia pseudopastoris* to the methylotrophic yeast genus *Komagataella*. Int. J. Syst. Evol. Microbiol. 55, 973–976 (2005). 15774694 10.1099/ijs.0.63491-0

[45] L. Bernauer, A. Radkohl, L. G. K. Lehmayer, A. Emmerstorfer-Augustin, *Komagataella phaffii* as emerging model organism in fundamental research. Front. Microbiol. 11, 607028 (2021). 33505376 10.3389/fmicb.2020.607028 PMC7829337

[46] G. Gellissen, G. Kunze, C. Gaillardin, J. Cregg, E. Berardi, M. Veenhuis, I. Van der Klei, New yeast expression platforms based on methylotrophic *Hansenula polymorpha* and *Pichia pastoris* and on dimorphic *Arxula adeninivorans* and *Yarrowia lipolytica* - A comparison. FEMS Yeast Res. 5, 1079–1096 (2005). 16144775 10.1016/j.femsyr.2005.06.004

[47] H. R. Bridges, L. Grgic, M. E. Harbour, J. Hirst, The respiratory complexes I from the mitochondria of two *Pichia* species. Biochem. J. 422, 151–159 (2009). 19459785 10.1042/BJ20090492

[48] H. R. Bridges, I. M. Fearnley, J. Hirst, The subunit composition of mitochondrial NADH: Ubiquinone oxidoreductase (Complex I) from *Pichia pastoris*. Mol. Cell. Proteomics 9, 2318–2326 (2010). 20610779 10.1074/mcp.M110.001255 PMC2953923

[49] I. Chung, J. J. Wright, H. R. Bridges, B. S. Ivanov, O. Biner, C. S. Pereira, G. M. Arantes, J. Hirst, Cryo-EM structures define ubiquinone-10 binding to mitochondrial complex I and conformational transitions accompanying Q-site occupancy. Nat. Commun. 13, 2758 (2022). 35589726 10.1038/s41467-022-30506-1 PMC9120487

[50] E. Laube, J. Meier-Credo, J. D. Langer, W. Kühlbrandt, Conformational changes in mitochondrial complex I of the thermophilic eukaryote *Chaetomium thermophilum*. Sci. Adv. 8, eadc9952 (2022). 36427319 10.1126/sciadv.adc9952 PMC9699679

[51] K. Jamali, L. Käll, R. Zhang, A. Brown, D. Kimanius, S. H. W. Scheres, Automated model building and protein identification in cryo-EM maps. Nature 628, 450–457 (2024). 38408488 10.1038/s41586-024-07215-4 PMC11006616

[52] J. Carroll, S. Ding, I. M. Fearnley, J. E. Walker, Post-translational modifications near the quinone binding site of mammalian complex I. J. Biol. Chem. 288, 24799–24808 (2013). 23836892 10.1074/jbc.M113.488106 PMC3750175

[53] S. Y. Kim, S. Yoon, Structural analysis of the YqeY proteins from *Campylobacter jejuni* and *Vibrio parahaemolyticus*. Biochem. Biophys. Res. Commun. 695, 149485 (2024). 38211535 10.1016/j.bbrc.2024.149485

[54] M. Deniziak, C. Sauter, H. D. Becker, C. A. Paulus, R. Giegé, D. Kern, *Deinococcus* glutaminyl-tRNA synthetase is a chimer between proteins from an ancient and the modern pathways of aminoacyl-tRNA formation. Nucleic Acids Res. 35, 1421–1431 (2007). 17284460 10.1093/nar/gkl1164 PMC1865053

[55] A. Nakamura, K. Sheppard, J. Yamane, M. Yao, D. Söll, I. Tanaka, Two distinct regions in *Staphylococcus aureus* GatCAB guarantee accurate tRNA recognition. Nucleic Acids Res. 38, 672–682 (2009). 19906721 10.1093/nar/gkp955 PMC2811023

[56] A. Padavannil, A. Murari, S.-K. Rhooms, E. Owusu-Ansah, J. A. Letts, Resting mitochondrial complex I from *Drosophila melanogaster* adopts a helix-locked state. eLife 12, e84415 (2023). 36952377 10.7554/eLife.84415 PMC10036122

[57] L. Zhou, M. Maldonado, A. Padavannil, F. Guo, J. A. Letts, Structures of *Tetrahymena*’s respiratory chain reveal the diversity of eukaryotic core metabolism. Science 7747, eabn7747 (2022). 10.1126/science.abn7747 PMC9169680 35357889

[58] B. S. Ivanov, H. R. Bridges, O. D. Jarman, J. Hirst, Structure of the turnover-ready state of an ancestral respiratory complex I. Nat. Commun. 15, 9340 (2024). 39472559 10.1038/s41467-024-53679-3 PMC11522691

[59] J. Abramson, J. Adler, J. Dunger, R. Evans, T. Green, A. Pritzel, O. Ronneberger, L. Willmore, A. J. Ballard, J. Bambrick, S. W. Bodenstein, D. A. Evans, C.-C. Hung, M. O’Neill, D. Reiman, K. Tunyasuvunakool, Z. Wu, A. Žemgulytė, E. Arvaniti, C. Beattie, O. Bertolli, A. Bridgland, A. Cherepanov, M. Congreve, A. I. Cowen-Rivers, A. Cowie, M. Figurnov, F. B. Fuchs, H. Gladman, R. Jain, Y. A. Khan, C. M. R. Low, K. Perlin, A. Potapenko, P. Savy, S. Singh, A. Stecula, A. Thillaisundaram, C. Tong, S. Yakneen, E. D. Zhong, M. Zielinski, A. Žídek, V. Bapst, P. Kohli, M. Jaderberg, D. Hassabis, J. M. Jumper, Accurate structure prediction of biomolecular interactions with AlphaFold 3. Nature 630, 493–500 (2024). 38718835 10.1038/s41586-024-07487-w PMC11168924

[60] E. V. Gavrikova, A. D. Vinogradov, Active/de-active state transition of the mitochondrial complex I as revealed by specific sulfhydryl group labeling. FEBS Lett. 455, 36–40 (1999). 10428467 10.1016/s0014-5793(99)00850-9

[61] A. Galkin, B. Meyer, I. Wittig, M. Karas, H. Schägger, A. Vinogradov, U. Brandt, Identification of the mitochondrial ND3 subunit as a structural component involved in the active/deactive enzyme transition of respiratory complex I. J. Biol. Chem. 283, 20907–20913 (2008). 18502755 10.1074/jbc.M803190200 PMC2475694

[62] J. Zhu, K. R. Vinothkumar, J. Hirst, Structure of mammalian respiratory complex I. Nature 536, 354–358 (2016). 27509854 10.1038/nature19095 PMC5027920

[63] J. N. Blaza, K. R. Vinothkumar, J. Hirst, Structure of the deactive state of mammalian respiratory complex I. Structure 26, 312–319 (2018). 29395787 10.1016/j.str.2017.12.014 PMC5807054

[64] A. A. Agip, J. N. Blaza, H. R. Bridges, C. Viscomi, S. Rawson, S. P. Muench, J. Hirst, Cryo-EM structures of complex I from mouse heart mitochondria in two biochemically defined states. Nat. Struct. Mol. Biol. 25, 548–556 (2018). 29915388 10.1038/s41594-018-0073-1 PMC6054875

[65] W. Zheng, P. Chai, J. Zhu, K. Zhang, High-resolution in situ structures of mammalian respiratory supercomplexes. Nature 14, 8248 (2024). 10.1038/s41586-024-07488-9 PMC11222160 38811722

[66] J. A. Letts, K. Fiedorczuk, L. A. Sazanov, The architecture of respiratory supercomplexes. Nature 537, 644–648 (2016). 27654913 10.1038/nature19774

[67] J. A. Letts, K. Fiedorczuk, G. Degliesposti, M. Skehel, L. A. Sazanov, Structures of respiratory supercomplex I+III_2_ reveal functional and conformational crosstalk. Mol. Cell 75, 1131–1146.e6 (2019). 31492636 10.1016/j.molcel.2019.07.022 PMC6926478

[68] A.-N. A. Agip, I. Chung, A. Sanchez-Martinez, A. J. Whitworth, J. Hirst, Cryo-EM structures of mitochondrial respiratory complex I from *Drosophila melanogaster*. eLife 12, e84424 (2023). 36622099 10.7554/eLife.84424 PMC9977279

[69] A. J. Y. Jones, J. N. Blaza, H. R. Bridges, B. May, A. L. Moore, J. Hirst, A self-assembled respiratory chain that catalyzes NADH oxidation by ubiquinone-10 cycling between complexI and the alternative oxidase. Angwe. Chem. Int. Ed. 55, 728–731 (2016). 10.1002/anie.201507332 PMC4954055 26592861

[70] O. Biner, J. G. Fedor, Z. Yin, J. Hirst, Bottom-up construction of a minimal system for cellular respiration and energy regeneration. ACS Synth. Biol. 9, 1450–1459 (2020). 32383867 10.1021/acssynbio.0c00110 PMC7611821

[71] H. Schagger, Supercomplexes in the respiratory chains of yeast and mammalian mitochondria. EMBO J. 19, 1777–1783 (2000). 10775262 10.1093/emboj/19.8.1777 PMC302020

[72] K. M. Davies, T. B. Blum, W. Kühlbrandt, Conserved in situ arrangement of complex I and III_2_ in mitochondrial respiratory chain supercomplexes of mammals, yeast, and plants. Proc. Natl. Acad. Sci. U.S.A. 115, 3024–3029 (2018). 29519876 10.1073/pnas.1720702115 PMC5866595

[73] M. Maldonado, Z. Fan, K. M. Abe, J. A. Letts, Plant-specific features of respiratory supercomplex I + III_2_ from *Vigna radiata*. Nat. Plants 9, 157–168 (2023). 36581760 10.1038/s41477-022-01306-8 PMC9873571

[74] M. Wu, J. Gu, R. Guo, Y. Huang, M. Yang, Structure of mammalian respiratory supercomplex I_1_III_2_IV_1_. Cell 167, 1598–1609.e10 (2016). 27912063 10.1016/j.cell.2016.11.012

[75] R. Guo, S. Zong, M. Wu, J. Gu, M. Yang, Architecture of human mitochondrial respiratory megacomplex I_2_III_2_IV_2_. Cell 170, 1247–1257 (2017). 28844695 10.1016/j.cell.2017.07.050

[76] N. Klusch, J. Senkler, Ö. Yildiz, W. Kühlbrandt, H.-P. Braun, A ferredoxin bridge connects the two arms of plant mitochondrial complex I. Plant Cell 33, 2072–2091 (2021). 33768254 10.1093/plcell/koab092 PMC8290278

[77] Z. He, M. Wu, H. Tian, L. Wang, Y. Hu, F. Han, J. Zhou, Y. Wang, L. Zhou, *Euglena*’s atypical respiratory chain adapts to the discoidal cristae and flexible metabolism. Nat. Commun. 15, 1628 (2024). 38388527 10.1038/s41467-024-46018-z PMC10884005

[78] A. B. Kotlyar, N. Borovok, NADH oxidation and NAD ^+^ reduction catalysed by tightly coupled inside-out vesicles from *Paracoccus denitrificans*. Eur. J. Biochem. 269, 4020–4024 (2002). 12180978 10.1046/j.1432-1033.2002.03091.x

[79] O. D. Jarman, J. Hirst, Membrane-domain mutations in respiratory complex I impede catalysis but do not uncouple proton pumping from ubiquinone reduction. PNAS Nexus 1, pgac276 (2022). 36712358 10.1093/pnasnexus/pgac276 PMC9802314

[80] Y. Liang, A. Plourde, S. A. Bueler, J. Liu, P. Brzezinski, S. Vahidi, J. L. Rubinstein, Structure of mycobacterial respiratory complex I. Proc. Natl. Acad. Sci. U.S.A. 120, e2214949120 (2023). 36952383 10.1073/pnas.2214949120 PMC10068793

[81] J. G. Fedor, A. J. Y. Jones, A. Di Luca, V. R. I. Kaila, J. Hirst, Correlating kinetic and structural data on ubiquinone binding and reduction by respiratory complex I. Proc. Natl. Acad. Sci. U.S.A. 114, 12737–12742 (2017). 29133414 10.1073/pnas.1714074114 PMC5715780

[82] J. Miehling, D. Goricanec, F. Hagn, A split-intein-based method for the efficient production of circularized nanodiscs for structural studies of membrane proteins. Chembiochem 19, 1927–1933 (2018). 29947468 10.1002/cbic.201800345

[83] K. Matsushita, T. Ohnishi, H. R. Kaback, NADH-ubiquinone oxidoreductases of the *Escherichia coli* aerobic respiratory chain. Biochemistry 26, 7732–7737 (1987). 3122832 10.1021/bi00398a029

[84] M. S. Sharpley, R. J. Shannon, F. Draghi, J. Hirst, Interactions between phospholipids and NADH: Ubiquinone oxidoreductase (Complex I) from bovine mitochondria. Biochemistry 45, 241–248 (2006). 16388600 10.1021/bi051809x

[85] D. Kimanius, L. Dong, G. Sharov, T. Nakane, S. H. W. Scheres, New tools for automated cryo-EM single-particle analysis in RELION-4.0. Biochem. J. 478, 4169–4185 (2021). 34783343 10.1042/BCJ20210708 PMC8786306

[86] A. Punjani, J. L. Rubinstein, D. J. Fleet, M. A. Brubaker, cryoSPARC: Algorithms for rapid unsupervised cryo-EM structure determination. Nat. Methods 14, 290–296 (2017). 28165473 10.1038/nmeth.4169

[87] J. Zivanov, T. Nakane, S. H. W. Scheres, A Bayesian approach to beam-induced motion correction in cryo-EM single-particle analysis. IUCrJ 6, 5–17 (2019). 10.1107/S205225251801463X PMC6327179 30713699

[88] S. Q. Zheng, E. Palovcak, J.-P. Armache, K. A. Verba, Y. Cheng, D. A. Agard, MotionCor2: Anisotropic correction of beam-induced motion for improved cryo-electron microscopy. Nat. Methods 14, 331–332 (2017). 28250466 10.1038/nmeth.4193 PMC5494038

[89] A. Rohou, N. Grigorieff, CTFFIND4: Fast and accurate defocus estimation from electron micrographs. J. Struct. Biol. 192, 216–221 (2015). 26278980 10.1016/j.jsb.2015.08.008 PMC6760662

[90] T. Bepler, A. Morin, M. Rapp, J. Brasch, L. Shapiro, A. J. Noble, B. Berger, Positive-unlabeled convolutional neural networks for particle picking in cryo-electron micrographs. Nat. Methods 16, 1153–1160 (2019). 31591578 10.1038/s41592-019-0575-8 PMC6858545

[91] J. Zivanov, T. Nakane, B. O. Forsberg, D. Kimanius, W. J. H. Hagen, E. Lindahl, S. H. W. Scheres, New tools for automated high-resolution cryo-EM structure determination in RELION-3. eLife 7, e42166 (2018). 30412051 10.7554/eLife.42166 PMC6250425

[92] E. F. Pettersen, T. D. Goddard, C. C. Huang, E. C. Meng, G. S. Couch, T. I. Croll, J. H. Morris, T. E. Ferrin, UCSF ChimeraX: Structure visualization for researchers, educators, and developers. Protein Sci. 30, 70–82 (2021). 32881101 10.1002/pro.3943 PMC7737788

[93] S. F. Altschul, W. Gish, W. Miller, E. W. Myers, D. J. Lipman, Basic local alignment search tool. J. Mol. Biol. 215, 403–410 (1990). 2231712 10.1016/S0022-2836(05)80360-2

[94] D. Liebschner, P. V. Afonine, M. L. Baker, G. Bunkoczi, V. B. Chen, T. I. Croll, B. Hintze, L. W. Hung, S. Jain, A. J. McCoy, N. W. Moriarty, R. D. Oeffner, B. K. Poon, M. G. Prisant, R. J. Read, J. S. Richardson, D. C. Richardson, M. D. Sammito, O. V. Sobolev, D. H. Stockwell, T. C. Terwilliger, A. G. Urzhumtsev, L. L. Videau, C. J. Williams, P. D. Adams, Macromolecular structure determination using X-rays, neutrons and electrons: Recent developments in Phenix. Acta Crystallogr. D Struct. Biol. 75, 861–877 (2019). 31588918 10.1107/S2059798319011471 PMC6778852

[95] P. Emsley, B. Lohkamp, W. G. Scott, K. Cowtan, Features and development of Coot. Acta Crystallogr. D Biol. Crystallogr. 66, 486–501 (2010). 20383002 10.1107/S0907444910007493 PMC2852313

[96] W. Tian, C. Chen, X. Lei, J. Zhao, J. Liang, CASTp 3.0: Computed atlas of surface topography of proteins. Nucleic Acids Res. 46, W363–W367 (2018). 29860391 10.1093/nar/gky473 PMC6031066

[97] G. Pintilie, K. Zhang, Z. Su, S. Li, M. F. Schmid, W. Chiu, Measurement of atom resolvability in cryo-EM maps with Q-scores. Nat. Methods 17, 328–334 (2020). 32042190 10.1038/s41592-020-0731-1 PMC7446556

[98] E. F. Pettersen, T. D. Goddard, C. C. Huang, G. S. Couch, D. M. Greenblatt, E. C. Meng, T. E. Ferrin, UCSF Chimera—A visualization system for exploratory research and analysis. J. Comput. Chem. 25, 1605–1612 (2004). 15264254 10.1002/jcc.20084

