# Supplementary material for: Global conformations of Pichia pastoris complex I are distinguished by the binding of a unique interdomain bridging subunit
Source: Sci Adv. 2025 Oct 1;11(40):eadz0693. doi: 10.1126/sciadv.adz0693 (PMC12487887; doi:10.1126/sciadv.adz0693)
Supplement: Supplementary file 1 — Figs. S1 to S11 Tables S1 to S7 [file sciadv.adz0693_sm.pdf]

Science Advances
AAAS

Supplementary Materials for

Global conformations of *Pichia pastoris* complex I are distinguished by the binding of a unique interdomain bridging subunit

Chris Seunggyu Lee *et al.*

Corresponding author: Daniel N. Grba, dng26@cam.ac.uk; Judy Hirst, jh480@cam.ac.uk

Sci. Adv. **11**, eadz0693 (2025)
DOI: 10.1126/sciadv.adz0693

This PDF file includes:

Figs. S1 to S11
Tables S1 to S7

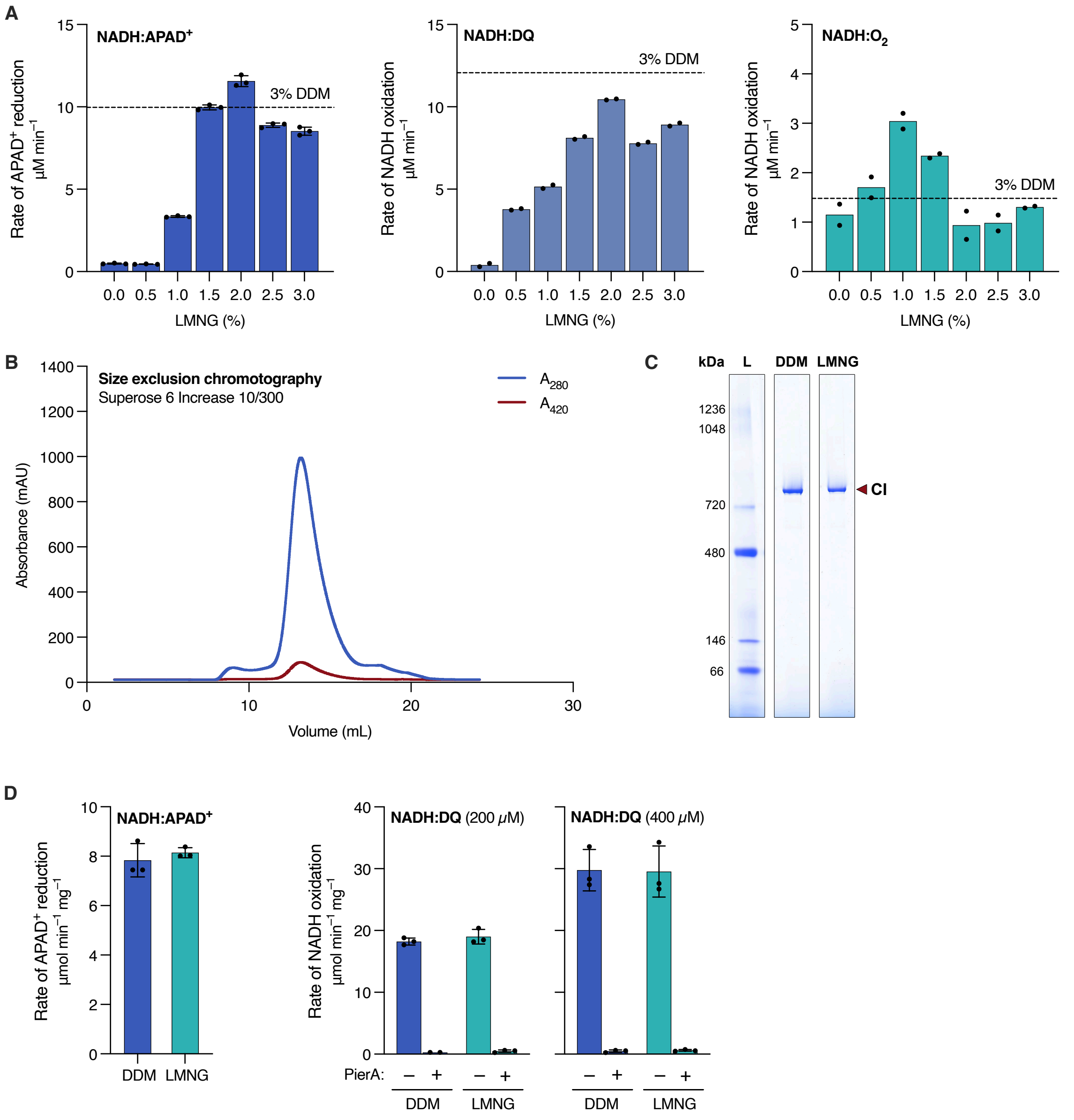

Fig. S1 | Solubilization and purification of complex I from *Pichia pastoris (Pp)*. (**A**) Solubilization trials of *Pp* membranes (15 mg mL^{-1}) in LMNG detergent, monitoring the rates of NADH:APAD$^+$, NADH:DQ and NADH:O_2 oxidoreduction. The mixtures were centrifuged and the same volume of sample added to each assay. NADH:O_2 activity assays were supplemented with 3 µM equine heart cytochrome *c* and indicate the presence of supercomplexes. Data from 3% DDM, as used in earlier preparations(*38*), are indicated, and 2% LMNG was selected for subsequent preparations. Assays were performed in 10 mM Tris-SO_4 (pH 7.5 at 32 ºC), 250 mM sucrose; see Methods for further experimental details. (**B**) Size-exclusion chromatogram of *Pp*-CI in 20 mM Na-MOPS (pH 7.4), 150 mM NaCl, 0.0375% LMNG buffer following purification on a Ni^{2+}-affinity column. The sample was injected onto a Superose 6 Increase 10/300 column and monomeric complex I elutes at ~11 mL. (**C**) BN-PAGE of *Pp*-CI purified in either DDM or LMNG. Both samples display a single band corresponding to the mass of complex I. *Pp*-CI (15 µg, 3 mg mL^{-1} in 1.5 M aminocaproic acid) was analyzed on a Native-PAGE 3–12% Bis-Tris gel (Novex) in Coomassie G-250 alongside a NativeMark™ unstained protein standard (Invitrogen) according to the manufacturer's instructions. (**D**) Comparison of the activities (NADH:APAD$^+$ and NADH:DQ oxidoreduction) of samples of *Pp*-CI isolated in either DDM (blue) or LMNG (green). The NADH:DQ activities are fully sensitive to piericidin A. Error bars indicate S.D., *n* = 3. See Methods for experimental details.

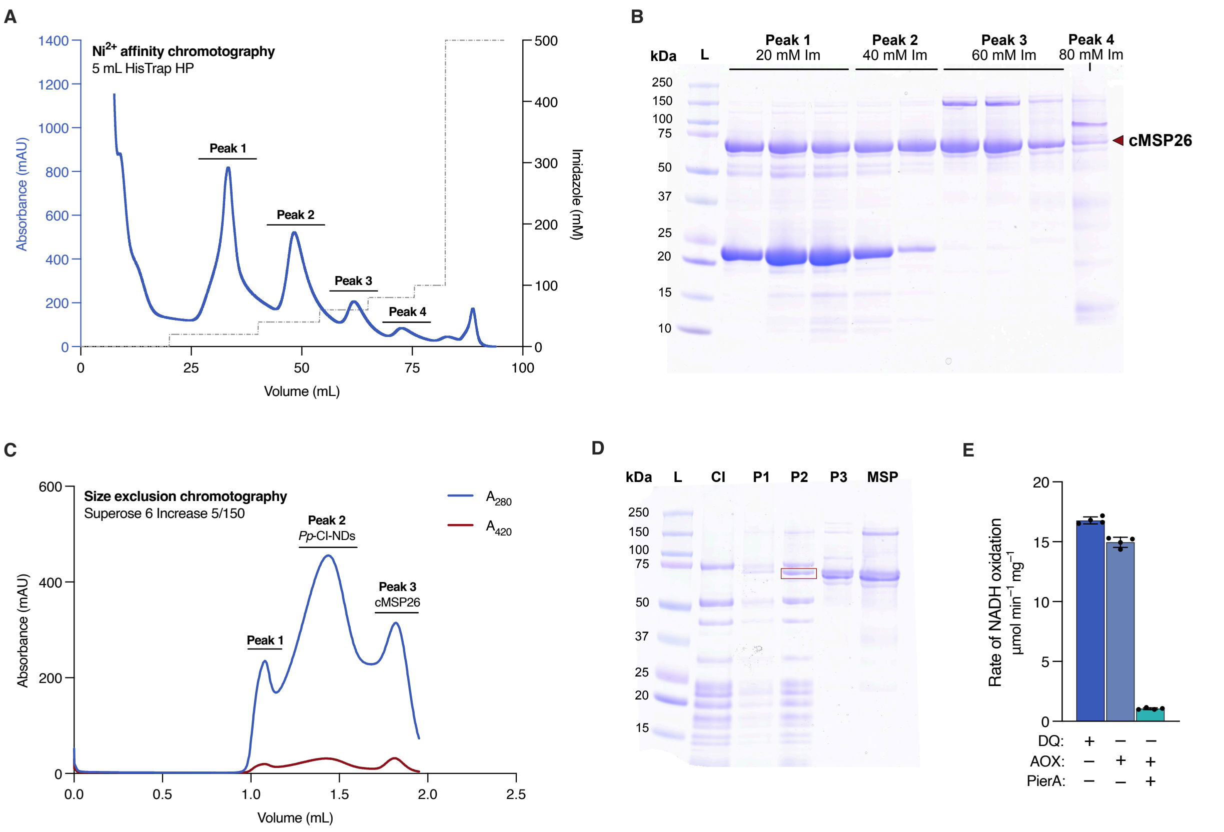

Fig. S2 | Reconstitution of *Pp*-CI into cMSP26 nanodiscs. (**A**) Elution of cMSP26 from the Ni^{2+}-affinity column monitored at 280 nm (blue). Step elution was carried out by increasing the imidazole concentration (grey). (**B**) SDS–PAGE analysis of peak fractions from the elution shown in **A** with the mass of cMSP26 indicated. Fractions eluting in 60 mM imidazole were collected. (**C**) Elution of *Pp*-CI-NDs from the Superose 6 Increase 5/150 column monitored at 280 nm (blue) and 420 nm (red). *Pp*-CI-NDs elute at 1.45 mL, ahead of CI-free NDs at 1.83 mL. (**D**) SDS–PAGE analysis of the three peak fractions (P1, P2, P3) from **C**. *Pp*-CI in LMNG (CI) and cMSP26 (MSP) were used as controls. The band from the cMSP26-NDs in P2 is highlighted in red, indicating the formation of *Pp*-CI-NDs. (**E**) Catalytic NADH oxidation assays on *Pp*-CI-NDs were measured with DQ, or with addition of 10 µg mL^{-1} AOX (alternative oxidase) to catalyze ubiquinol oxidation with O_2, confirming that the CI is functional. PierA is the complex I inhibitor piericidin A. Error bars indicate S.D., $n = 4$. See Methods for experimental details. In B and D, the samples were analyzed on 4–12% Bolt™ Bis-Tris Plus Mini Protein Gels according to the manufacturer's instructions and visualized with Coomassie R-250.

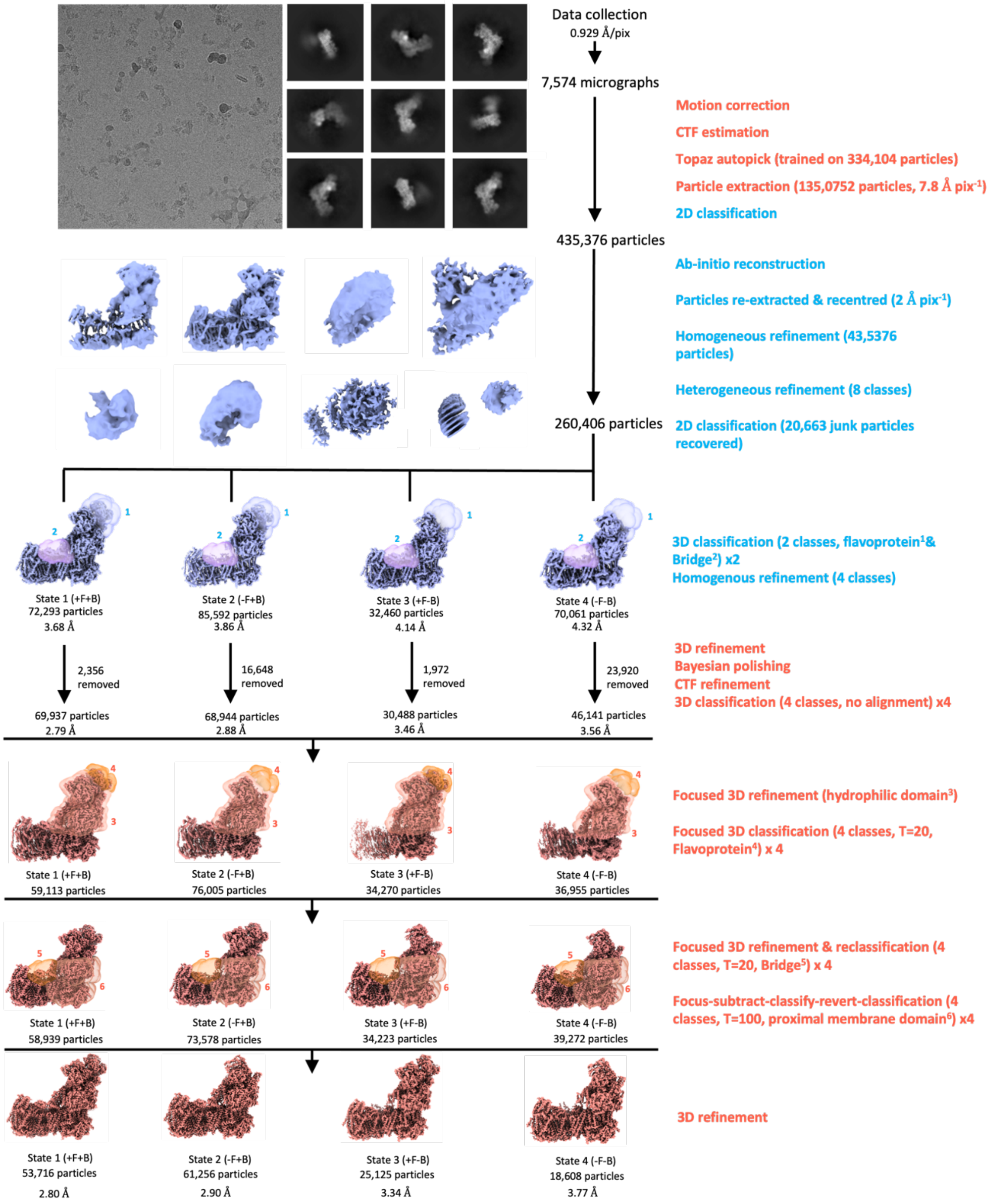

Fig. S3 | CryoEM data processing scheme for *Pp*-CI-NDs. The flowchart shows the pipeline employed to obtain the final four maps for the *Pp*-CI-NDs. An example micrograph and 2D class averages, along with cryoEM density maps are shown. Masks used for each step are shown as semi-transparent volumes and are numbered by the targeted regions described in the right-hand labels, marked with the corresponding numerical superscript. The volumes and steps are colored according to the software used (red: *RELION* v4.0(*85*), blue: *CryoSPARC* v3.3.2(*86*)).

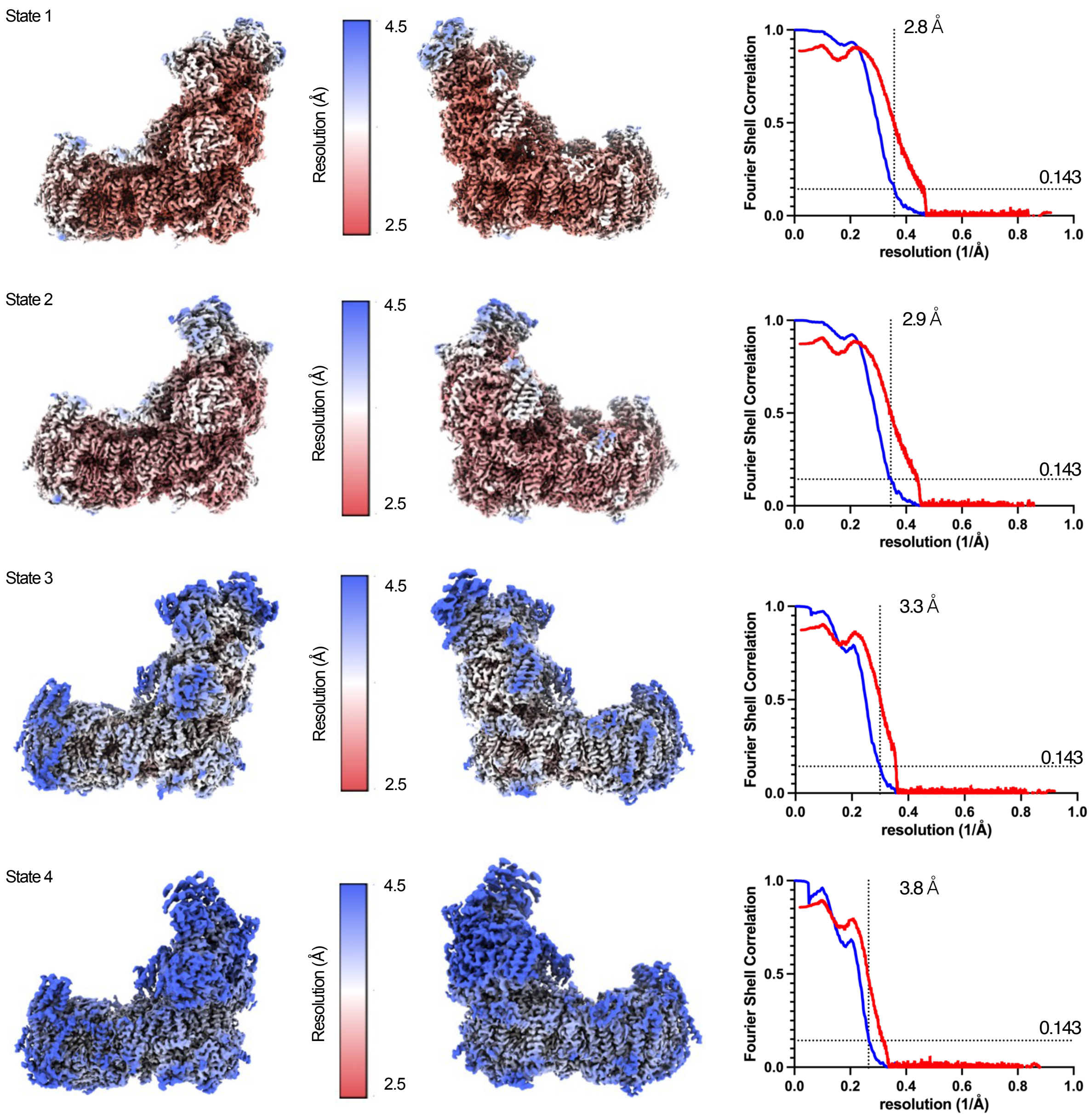

Fig. S4 | Local and global resolutions of the final four states of *Pp*-CI. Local resolutions (left) were calculated by *RELION* v4.0 and colored as shown in the key. Global Fourier Shell Correlation (FSC) scores (right) were calculated in *RELION* v4.0 (blue) and plotted along with model-map FSCs calculated using the validation suite in *Phenix* v1.21-5207 (red)(*94*). Resolutions are estimated by the FSC 0.143 criterion. The model-map FSCs are beyond the reported resolutions for all states, indicating a good fit of models to the cryoEM density.

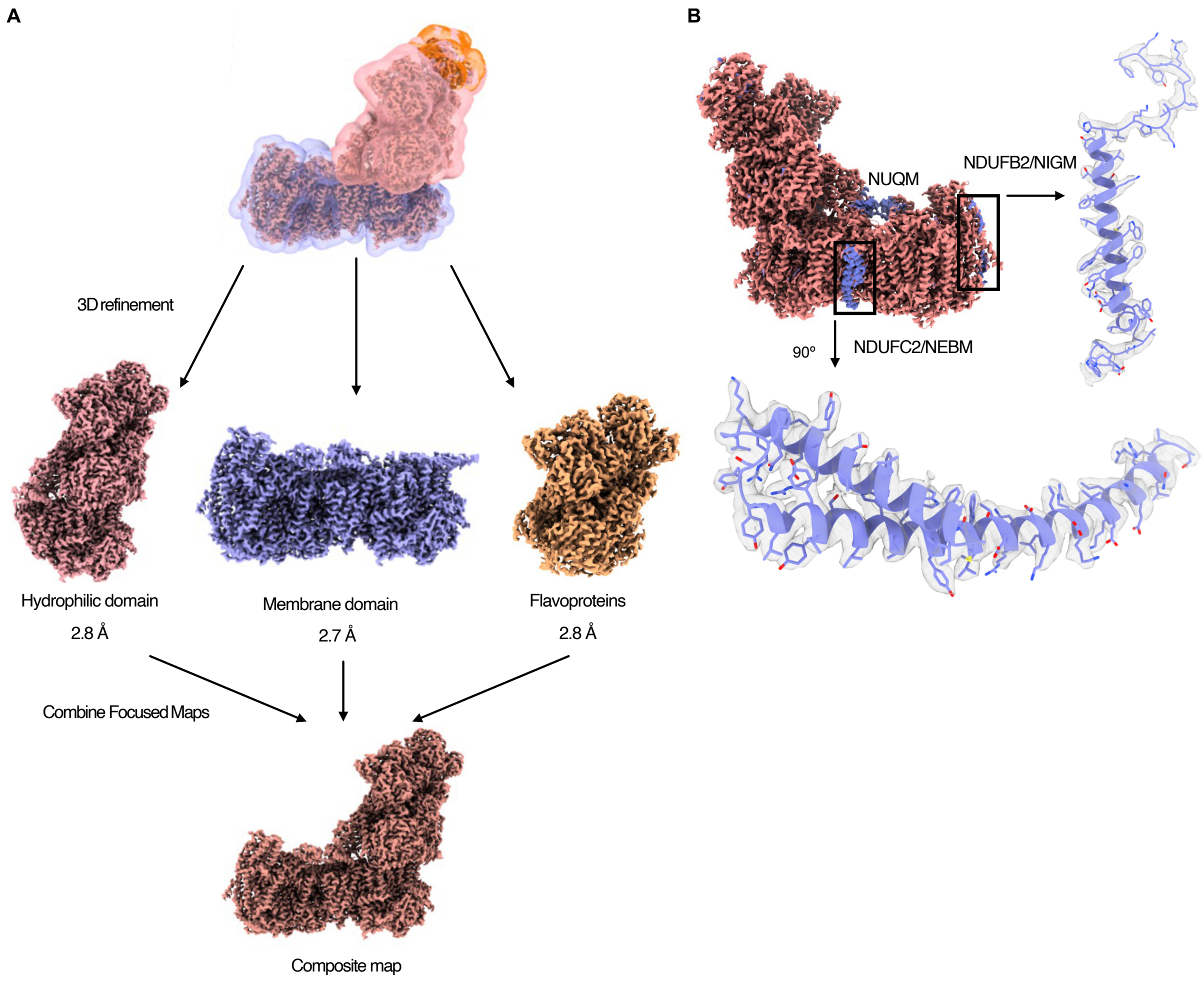

Fig. S5 | Generation of the composite map and identification of unknown subunit densities. (**A**) A flowchart showing the steps taken to generate the consensus composite map. The 2.8-Å resolution state 1 map from *RELION* v4.0 was divided into the membrane domain, the hydrophilic domain and the flavoprotein sub-domain of the hydrophilic domain, each of which was refined separately. The refined maps were then combined to produce a composite map using *Phenix* v1.21-5207. (**B**) The cryoEM density of the 2.8-Å resolution *Pp*-CI state 1 map obtained from *RELION* v4.0 showing the regions of assigned density from known subunits (red) and hitherto unknown subunits (blue). The latter include the novel NUQM subunit and also the homologous NDUFB2/NIGM and NDUFC2/NEBM subunits (not detected previously in *Pp*-CI). The sequences for these three subunits were predicted by *ModelAngelo*(*51*) using the composite map resulting in *BLASTp*(*93)* hits that correlated with sequences in the *Pp* genome (accession codes CAH2448636, XP_002492753 and XP_002490382). Insets show cryoEM densities of the homologous subunits with these sequences modelled (the cryoEM density for NUQM is shown in the main text).

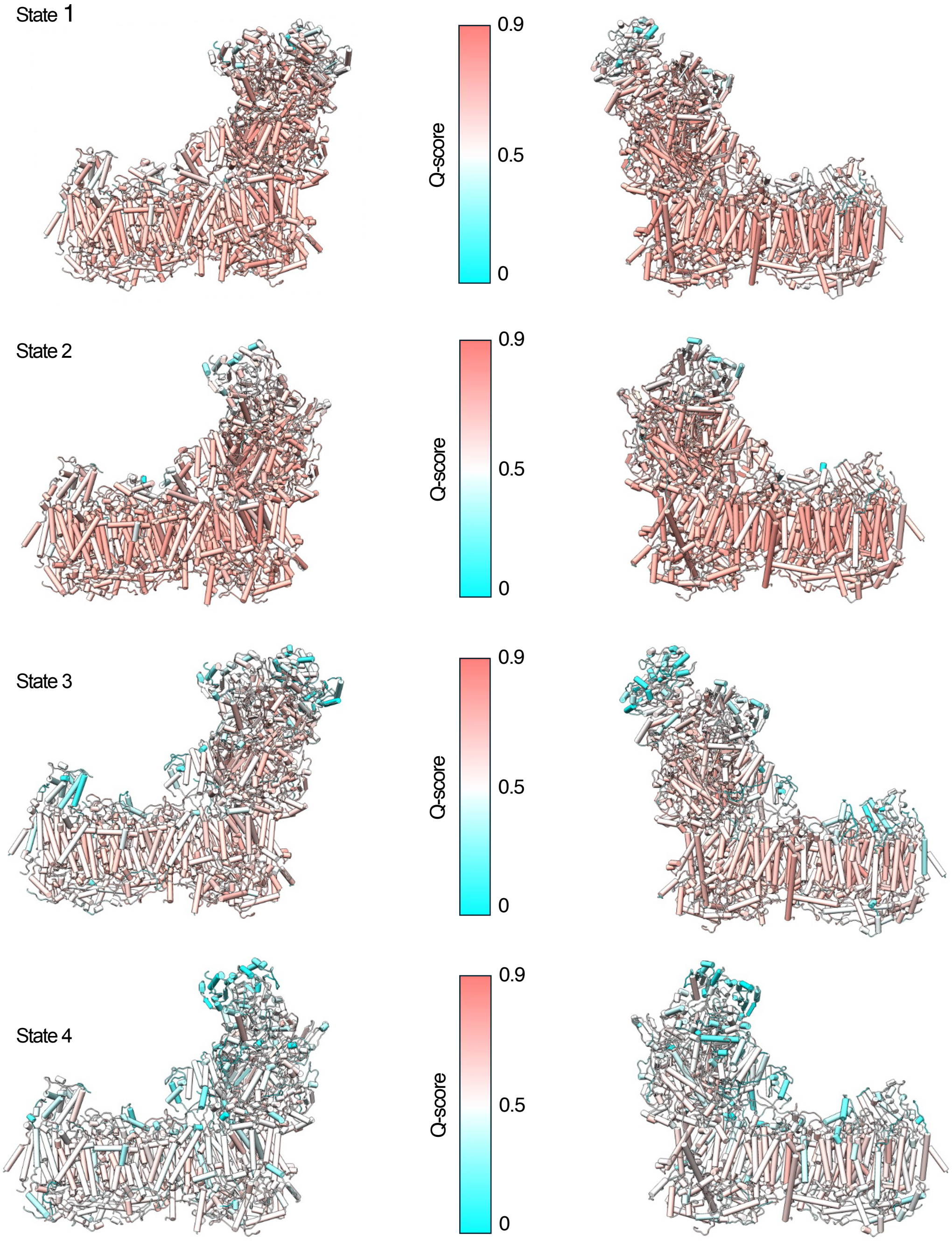

Fig. S6 | Per-residue *Q*-scores of *Pp*-CI maps and models. *Q*-scores(*97*) were calculated using the MapQ Plugin for states 1–4 and mapped out using *UCSF Chimera* v1.13(*98*). Colors of the models are indicated by keys, showing poor resolutions (low *Q*-scores) in cyan and high resolutions (high Q-scores) in pink.

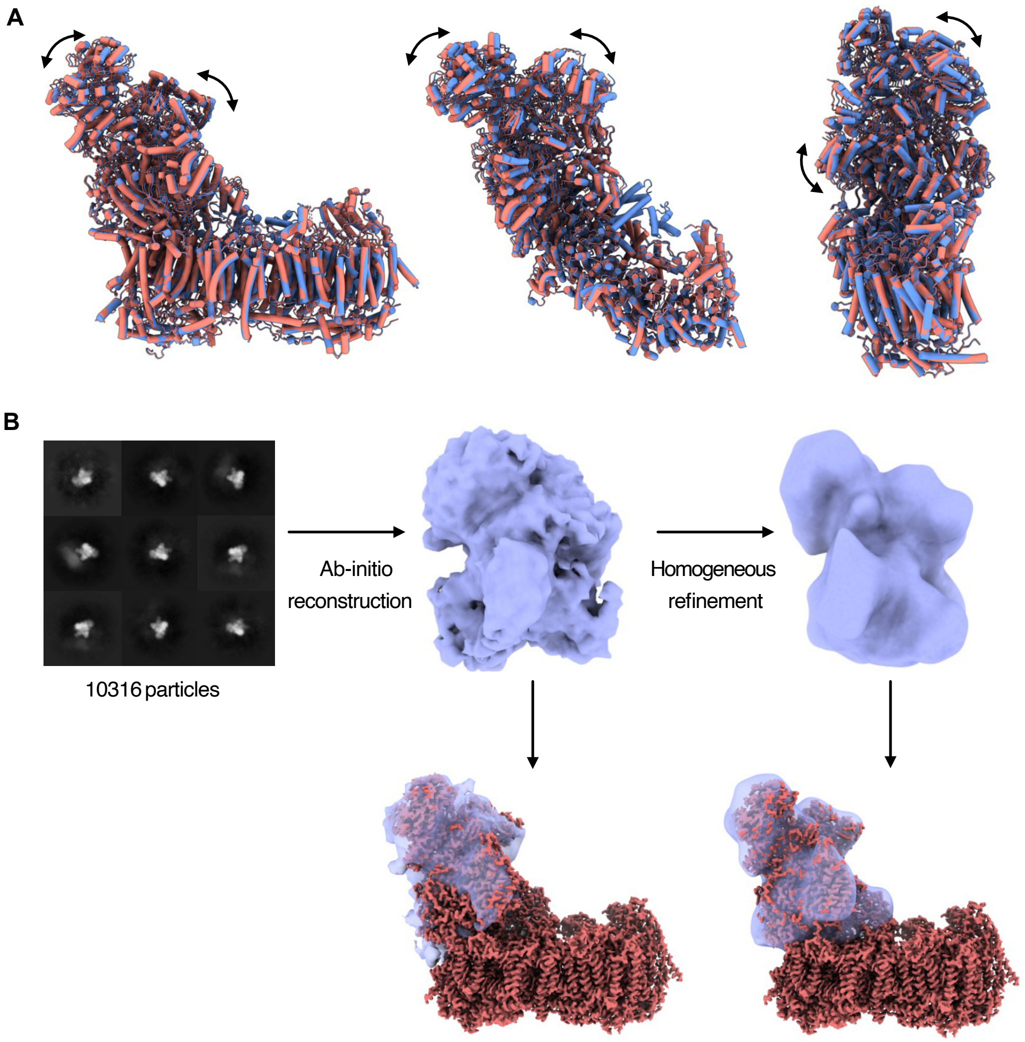

Fig. S7 | Changes in global protein conformation between the closed and open states, and the isolated hydrophilic domain identified in cryoEM classification. (**A**)The NUQM-bound (blue) and NUQM-free (red) states of *Pp*-CI are aligned based on the membrane-domain core subunits (ND1, ND2, ND3, ND4, ND4L, ND5 and ND6). The transition between the states involves a twisting motion, characterized by superior (upward) and lateral movement of the hydrophilic arm relative to the membrane arm, as indicated by the black arrows. (**B**) 10,316 particles that resembled the isolated hydrophilic domain were selected from 2D classification, subjected to ab-initio reconstruction and then homogeneous refinement. Generated hydrophilic domain volumes (blue surfaces) were docked onto the *Pp*-CI consensus state 1 map (red surfaces) using the fit map tool in *UCSF Chimera X*(*92)*, demonstrating good agreement with the entire hydrophilic domain.

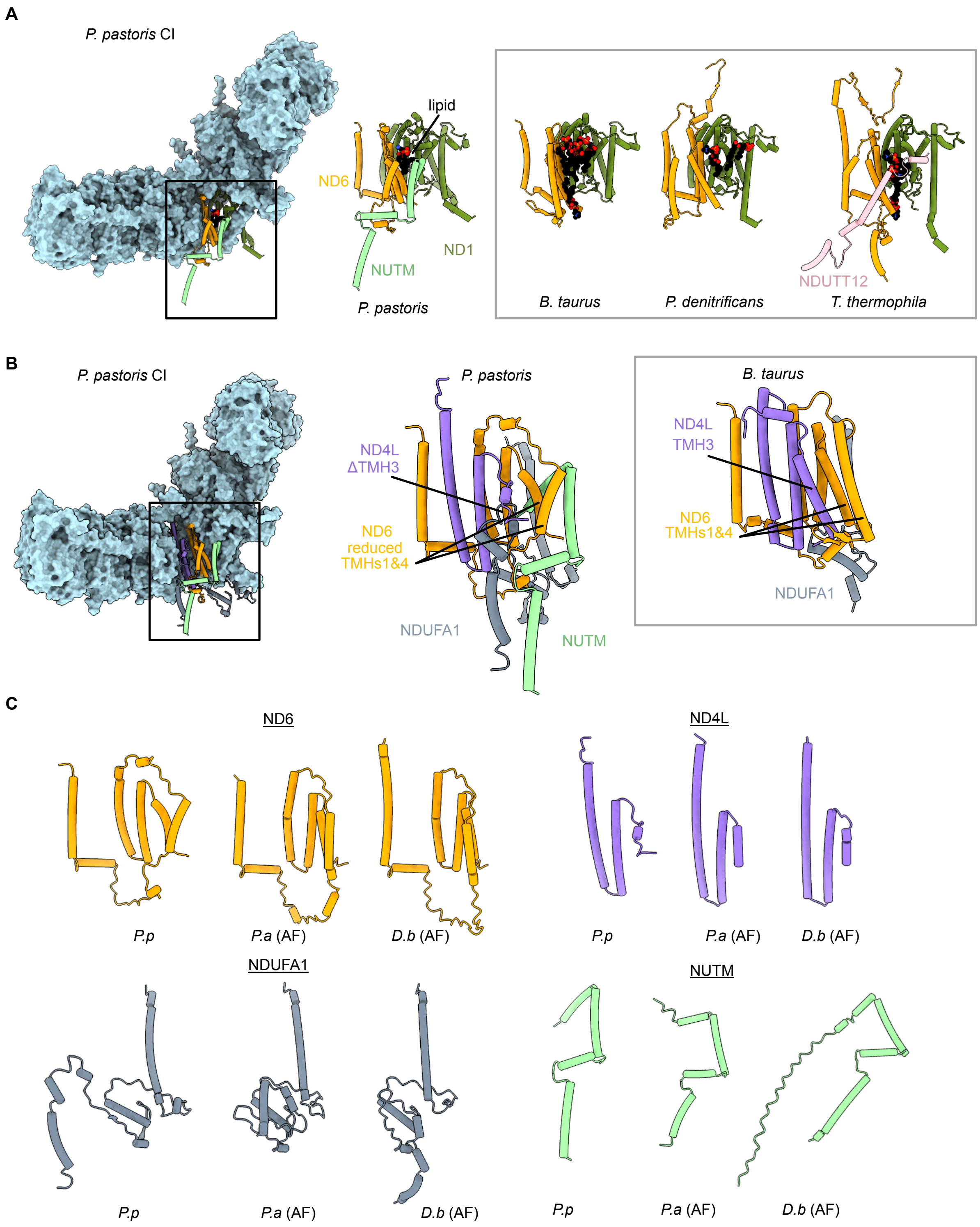

Fig. S8 | Structural adaptations around NUTM. (**A**) NUTM occupies a region that is associated with extensive lipid binding in other species (grey box). *Tetrahymena thermophilus* contains a unique NDUTT12 subunit that occupies a similar conformation and appears to trap a lipid in the same way as NUTM. (**B**) The adaptations of the core subunits ND4L and ND6 that interact with NUTM and NDUFA1 relative to in the mammalian enzyme (inset). (**C**) *Pichia pastoris* (*P.p*) subunits ND6, ND4L, NDUFA1 and NUTM and the respective *AlphaFold3* (AF)(*59*) predictions for the subunits from *Pichia angusta* (*P.a*, or *Ogataea polymorpha*) and *Dekkera bruxellensis* (*D.b* or *Brettanomyces bruxellensis*) that were highlighted as having NUTM subunit homologues.

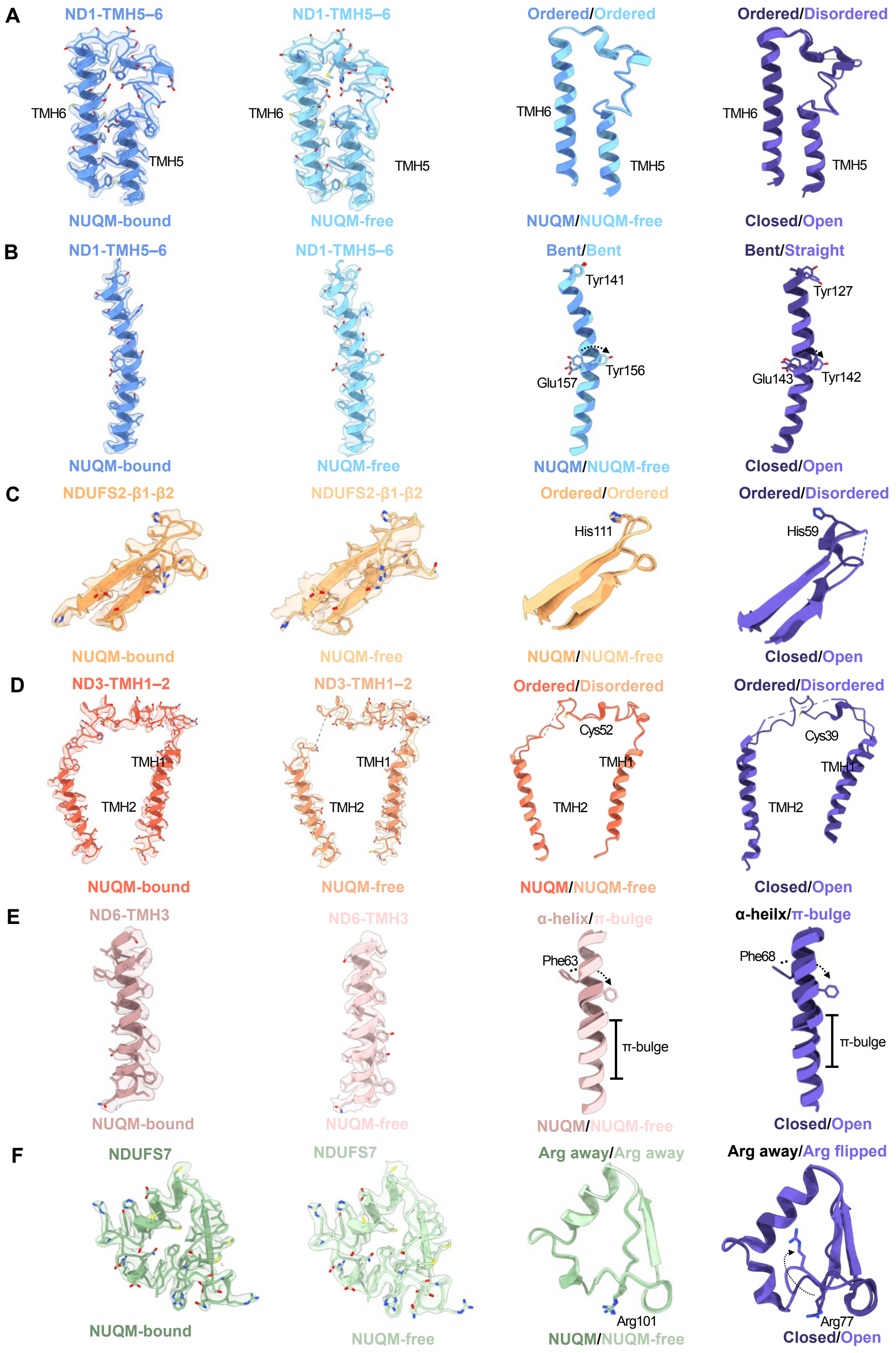

Fig. S9 | Elements of the active/closed and deactive/open transition in *Pp*-CI. The hallmarks of the mammalian-type active/closed to deactive/open transition are inspected in *Pp*-CI along with their respective map densities. Relevant residues are labelled, and subunits are colored as follows: (**A**–**B**) ND1; blue, (**C**) NDUSF2; orange, (**D**) ND3; red, (**E**) ND6; pink, (**F**) NDUSF7; green. All have the NUQM-free state shown in lighter shades. The columns show: first, the *Pp*-CI NUQM-bound state; second, the *Pp*-CI NUQM-free state; third, overlay of *Pp*-CI NUQM-bound and -free; fourth, reference bovine CI models in purple [PDB:8Q48 (closed/active), 8Q49 (open2/deactive)].

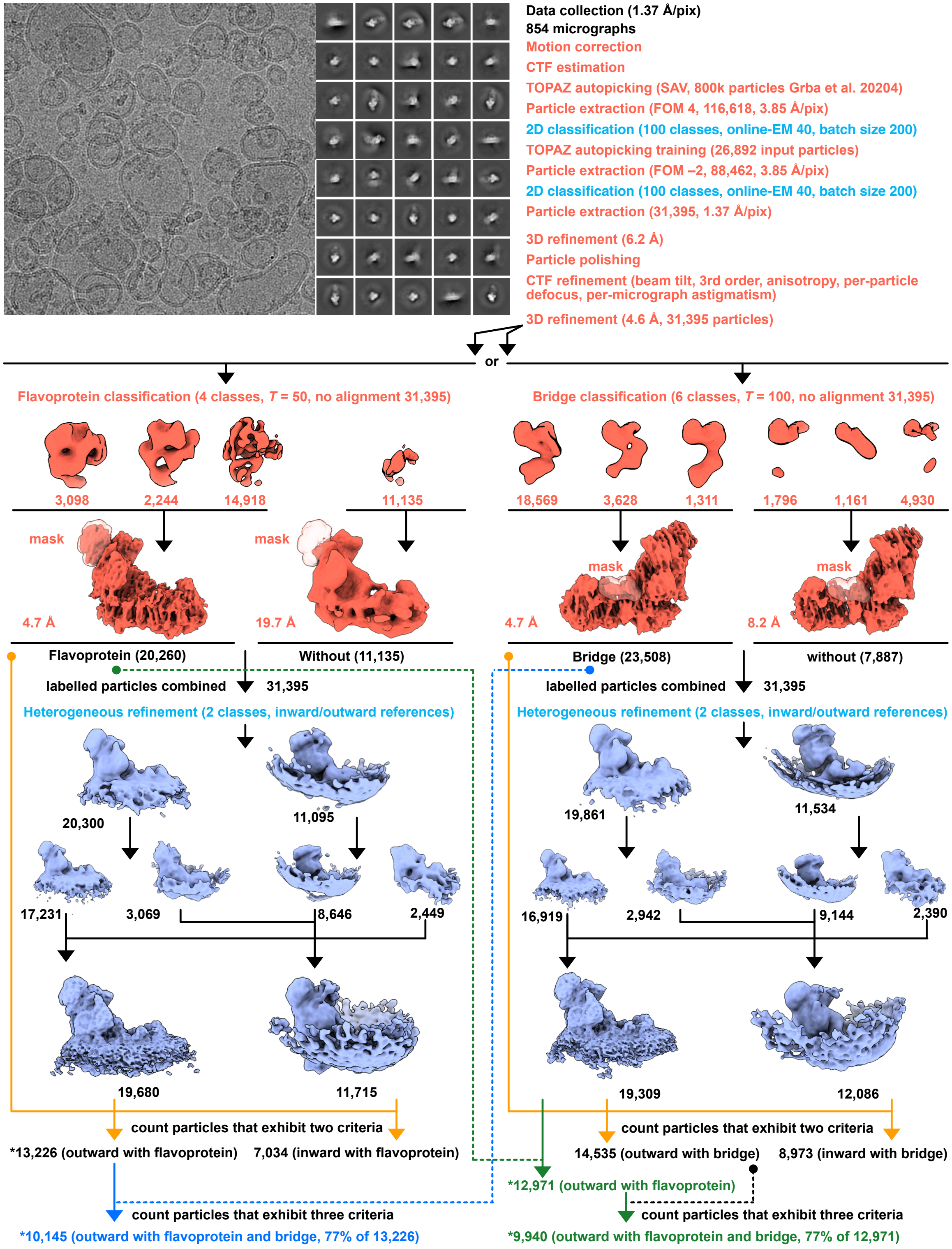

Fig. S10 | CryoEM data processing scheme for *Pp*-CI in proteoliposomes. The pipelines used to obtain cryoEM density maps for *Pp*-CI in PLs, and to classify the particles according to the presence of the flavoprotein (left, red) or bridge/NUQM (right, red), and to their orientation (outward/inward facing, determined separately in each pipeline, blue). Particles were labelled at each stage to enable the number defined by multiple criteria to be counted. An example micrograph and 2D class averages are shown. The colors represent the software used (red: *RELION* v4.0, blue: *CryoSPARC* v3.3.2). Asterisks (*) indicate biochemically measured species.

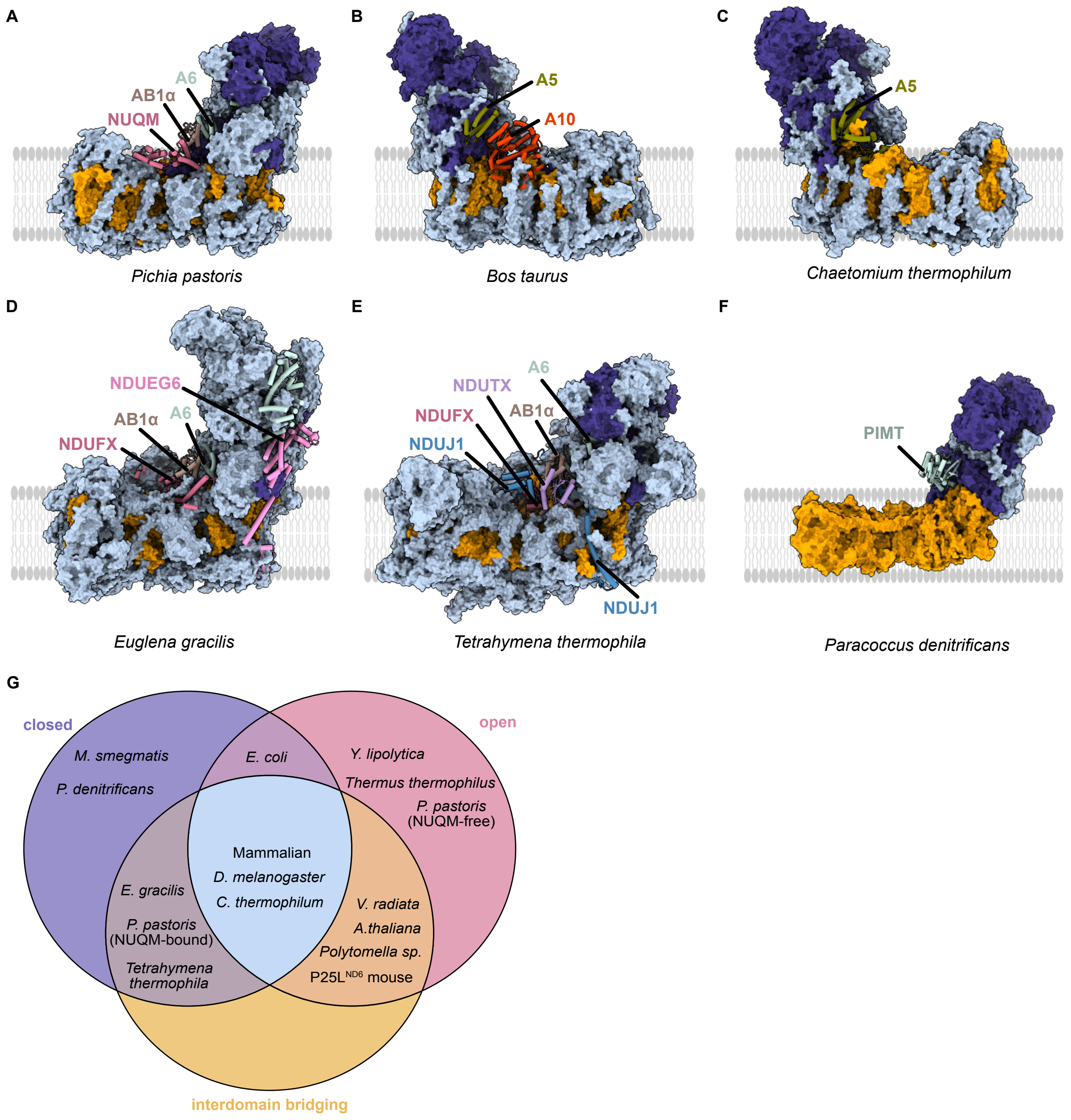

Fig. S11 | A comparison of complex I structures from species that are known to adopt or stabilize the closed state. (**A**) The multi-subunit bridging interaction between the hydrophilic and membrane domains in *Pichia pastoris* CI. (**B**) The bridging interaction of NDUFA5 and NDUFA10 in *Bos taurus* CI (PDB: 8Q48). (**C**) The single subunit (NDUFA5) bridge in *Chaetomium thermophilum* CI (PDB: 7ZMB). (**D**) The multi-subunit bridging and encapsulation of *Euglena gracilis* CI (PDB: 8J9I). (**E**) The multi-subunit bridging and encapsulation of *Tetrahymena thermophila* CI (PDB: 7TGH). (**F**) The single subunit (PIMT, protein L-isoaspartyl-O-methyltransferase) near the domain interface of *Paracoccus denitrificans* CI (PDB: 8QBY). Core membrane-domain subunits are in solid orange, core hydrophilic-domain subunits are in dark blue, and supernumerary subunits are in light blue unless otherwise highlighted. (**G**) A Venn diagram of complex I structures determined for various species demonstrates that the existence of closed states does not directly correlate with the existence of a bridge between the membrane and hydrophilic domains.

Table S1 | CryoEM data collection, refinement and validation statistics.

**Data collection and processing**				
Nominal magnification	130,000			
Voltage (kV)	300			
Electron exposure (e^{-} Å^{-2})	48			
Targeted defocus range (µm)	-0.6 to -1.8			
Calibrated pixel size (Å)	0.929			
Symmetry imposed	*C*1			
EMPIAR code	EMPIAR-12583			
Initial particle images (no.)	1,350,752			
State and model (PDB/EMDB codes)	**state 1 (9IHR/EMD-52878)**	**state 2 (9IHQ/EMD-52877)**	**state 3 (9IHP/EMD-52876)**	**state 4 (9IHO/EMD-52875)**
Final particle images (no.)	53,716	61,256	25,125	18,608
Map resolution (Å) (FSC 0.143)	2.8	2.9	3.3	3.8
Map resolution range (Å)	2.5–5.4	2.6–5.8	3.0–7.6	3.4–8.2
Map sharpening (RELION) *B* factor (Å^2)	−1	−10	−10	−29
**Refinement**				
Initial model used	ModelAngelo	9IHR	9IHR	9IHR
Model resolution (Å) (FSC 0.5)	2.8	2.9	3.3	3.8
Model composition				
Nonhydrogen atoms	70,216	64,071	68,464	61,836
Protein residues	8516	7729	8315	7472
Ligands	61	58	58	52
B factors mean (Å^2)				
Protein	40.36	37.05	58.18	35.56
Ligand	55.34	53.84	70.04	40.84
RMS deviations				
Bond lengths (Å)	0.002	0.003	0.006	0.004
Bond angles (°)	0.425	0.475	0.585	0.510
Validation				
MolProbity score	1.61	1.75	2.33	2.20
Clashscore	5.61	6.31	9.02	9.04
Rotamer outliers (%)	2.00	2.25	5.24	3.56
Cß outliers (%)	0.00	0.00	0.00	0.00
Ramachandran plot				
Favored (%)	97.67	97.32	95.67	95.64
Allowed (%)	2.30	2.64	4.26	4.32
Outliers (%)	0.04	0.04	0.07	0.04
Rama-Z (Ramachandran plot Z-score, RMSD)				
Whole	0.68 (0.09)	0.09 (0.09)	-0.88 (0.09)	-0.63 (0.10)
Helix	0.93 (0.08)	0.32 (0.08)	-0.39 (0.08)	-0.17 (0.08)
Sheet	0.60 (0.27)	0.01 (0.29)	-0.58 (0.27)	-0.46 (0.30)
Loop	-0.03 (0.11)	-0.10 (0.11)	-0.64 (0.11)	-0.55 (0.11)

Table S2 | Summary of the model built for state 1 of *Pp*-CI.

Subunit	Alternative names	Chain	Total residues	Modelled residues	Modelled cofactors and modifications
**NUBM**	51 kDa, NDUFV1	F	473	21–473	FMN, 4Fe4S
**NUHM**	24 kDa, NDUFV2	E	241	27–209	2Fe2S
**NUAM**	75 kDa, NDUFS1	G	726	25–720	2Fe2S, 2 x 4Fe4S
**NUCM**	49 kDa, NDUFS2	D	482	32–482	Dimethyl-Arg137
**NUGM**	30 kDa, NDUFS3	C	289	42–281	
**NUKM**	PSST, NDUFS7	B	204	30–204	4Fe4S
**NUIM**	TYKY, NDUFS8	I	222	31–222	2 x 4Fe4S
**NU1M**	ND1	H	353	1–353	N-formyl
**NU2M**	ND2	N	523	1–347, 365–523	N-formyl
**NU3M**	ND3	A	141	1–141	N-formyl
**NU4M**	ND4	M	491	1–491	N-formyl
**NULM**	ND4L	K	82	1–80	N-formyl
**NU5M**	ND5	L	642	1–642	N-formyl
**NU6M**	ND6	J	161	1–161	N-formyl
**NUYM**	18 kDa, NDUFS4	Q	159	35–146	
**NIPM**	15 kDa, NDUFS5	e	106	2–106	2 x Cys–Cys
**NUMM**	13 kDa, NDUFS6	R	139	13–136	Zn^{2+}
**NIMM**	MWFE, NDUFA1	a	150	1–149	
**NI8M**	B8, NDUFA2	S	89‡	1–89	-Met, N-acetyl
**NI9M**	B9, NDUFA3	b	79	2–79	
**NUFM**	B13, NDUFA5	V	134	9–134	
**NB4M**	B14, NDUFA6	W	122	2–102, 109–122	
**NUZM**	B14.5a, NDUFA7	c	181‡	1–181	-Met, N-acetyl
**NUPM**	PGIV, NDUFA8	X	183‡	1–183	-Met, N-acetyl, 3 x Cys–Cys
**NUEM**	39 kDa, NDUFA9	P	384	8–375	NADPH
**NUXM**	—	O	192	1–192	N-acetyl
**NUJM**	B14.7, NDUFA11	Y	216	12–216	
**N7BM**	B17.2, NDUFA12	q	139‡	1–139	-Met, N-acetyl
**NB6M**	B16.6, NDUFA13	Z	146‡	1–141	-Met, N-acetyl
**ACPM1**	SDAPα, NDUFAB1α	T	138	44–138	4'-phosphopantethine + 3-hydroxytetradecanoate
**ACPM2**	SDAPβ, NDUFAB1β	U	130	43–130	
**NUTM**	—	f	86	2–79	
**NIGM**	AGGG, NDUFB2*	j	59	7–59	
**NB2M**	B12, NDUFB3	k	61	2–46	
**NB5M**	B15, NDUFB4	m	81	5–81	
**NUSM**	SGDH, NUNM, NDUFB5*	h	182	52–182	
**NUUM**	B17, NDUFB6†	i	74	4–72	
**NB8M**	B18, NDUFB7	o	87	2–81	Cys–Cys
**NIAM**	ASHI, NDUFB8	l	156	25–156	
**NI2M**	B22, NDUFB9	n	111	7–111	
**NIDM**	PDSW, NDUFB10	p	92	3–92	Cys–Cys
**NESM**	ESSS, NDUFB11	g	239	25–46,73–105, 132–234	
**NUQM**	—	1	190	21–189	
**NEBM**	B14.5b, NDUFC2*	d	78	1–75	

*Structural homologue, †positional homologue ‡-Met, +N-acetyl group.

Table S3 | Summary of the model built for state 2 of *Pp*-CI.

Subunit	Alternative names	Chain	Total residues	Modelled residues	Modelled cofactors and modifications
**NUAM**	75 kDa, NDUFS1	G	726	55–75, 85–91, 101–146, 156–189, 212–669, 679–704	2Fe2S, 2 x 4Fe4S
**NUCM**	49 kDa, NDUFS2	D	482	32–482	Dimethyl-Arg137
**NUGM**	30 kDa, NDUFS3	C	289	42–281	
**NUKM**	PSST, NDUFS7	B	204	30–204	4Fe4S
**NUIM**	TYKY, NDUFS8	I	222	31–222	2 x 4Fe4S
**NU1M**	ND1	H	353	1–353	N-formyl
**NU2M**	ND2	N	523	1–347, 365–523	N-formyl
**NU3M**	ND3	A	141	1–141	N-formyl
**NU4M**	ND4	M	491	1–491	N-formyl
**NULM**	ND4L	K	82	1–80	N-formyl
**NU5M**	ND5	L	642	1–642	N-formyl
**NU6M**	ND6	J	161	1–161	N-formyl
**NUYM**	18 kDa, NDUFS4	Q	159	35–60, 72–131	
**NIPM**	15 kDa, NDUFS5	e	106	2–106	2 x Cys–Cys
**NUMM**	13 kDa, NDUFS6	R	139	13–136	Zn^{2+}
**NIMM**	MWFE, NDUFA1	a	150	1–149	
**NI8M**	B8, NDUFA2	S	89‡	1–89	-Met, N-acetyl
**NI9M**	B9, NDUFA3	b	79	2–79	
**NUFM**	B13, NDUFA5	V	134	9–134	
**NB4M**	B14, NDUFA6	W	122	2–102, 109–122	
**NUZM**	B14.5a, NDUFA7	c	181‡	1–48, 70–181	-Met, N-acetyl
**NUPM**	PGIV, NDUFA8	X	183‡	1–183	-Met, N-acetyl, 3 x Cys–Cys
**NUEM**	39 kDa, NDUFA9	P	384	8–375	NADPH
**NUXM**	—	O	192	1–192	N-acetyl
**NUJM**	B14.7, NDUFA11	Y	216	12–216	
**N7BM**	B17.2, NDUFA12	q	139‡	1–139	-Met, N-acetyl
**NB6M**	B16.6, NDUFA13	Z	146‡	1–141	-Met, N-acetyl
**ACPM1**	SDAPα, NDUFAB1α	T	138	44–138	4'-phosphopantethine + 3-hydroxytetradecanoate
**ACPM2**	SDAPβ, NDUFAB1β	U	130	43–130	
**NUTM**	—	f	86	2–79	
**NIGM**	AGGG, NDUFB2*	j	59	7–59	
**NB2M**	B12, NDUFB3	k	61	2–46	
**NB5M**	B15, NDUFB4	m	81	5–81	
**NUSM**	SGDH, NUNM, NDUFB5*	h	182	52–182	
**NUUM**	B17, NDUFB6†	i	74	4–72	
**NB8M**	B18, NDUFB7	o	87	2–81	Cys–Cys
**NIAM**	ASHI, NDUFB8	l	156	25–156	
**NI2M**	B22, NDUFB9	n	111	7–111	
**NIDM**	PDSW, NDUFB10	p	92	3–92	Cys–Cys
**NESM**	ESSS, NDUFB11	g	239	25–46,73–105, 132–234	
**NUQM**	—	1	190	21–189	
**NEBM**	B14.5b, NDUFC2*	d	78	1–75	

*Structural homologue, †positional homologue ‡-Met, +N-acetyl group.

Table S4 | Summary of the model built for state 3 of *Pp*-CI.

Subunit	Alternative names	Chain	Total residues	Modelled residues	Modelled cofactors and modifications
**NUBM**	51 kDa, NDUFV1	F	473	21–473	FMN, 4Fe4S
**NUHM**	24 kDa, NDUFV2	E	241	27–209	2Fe2S
**NUAM**	75 kDa, NDUFS1	G	726	25–720	2Fe2S, 2 x 4Fe4S
**NUCM**	49 kDa, NDUFS2	D	482	39–44, 49–482	Dimethyl-Arg137
**NUGM**	30 kDa, NDUFS3	C	289	42–281	
**NUKM**	PSST, NDUFS7	B	204	30–204	4Fe4S
**NUIM**	TYKY, NDUFS8	I	222	31–222	2 x 4Fe4S
**NU1M**	ND1	H	353	1–353	N-formyl
**NU2M**	ND2	N	523	1–347, 365–523	N-formyl
**NU3M**	ND3	A	141	1–57, 62–141	N-formyl
**NU4M**	ND4	M	491	1–491	N-formyl
**NULM**	ND4L	K	82	1–80	N-formyl
**NU5M**	ND5	L	642	1–642	N-formyl
**NU6M**	ND6	J	161	1–74, 81–161	N-formyl
**NUYM**	18 kDa, NDUFS4	Q	159	35–146	
**NIPM**	15 kDa, NDUFS5	e	106	2–106	2 x Cys–Cys
**NUMM**	13 kDa, NDUFS6	R	139	13–136	Zn^{2+}
**NIMM**	MWFE, NDUFA1	a	150	1–149	
**NI8M**	B8, NDUFA2	S	89‡	1–89	-Met, N-acetyl
**NI9M**	B9, NDUFA3	b	79	2–79	
**NUFM**	B13, NDUFA5	V	134	9–134	
**NB4M**	B14, NDUFA6	W	122	2–102, 109–122	
**NUZM**	B14.5a, NDUFA7	c	181‡	1–181	-Met, N-acetyl
**NUPM**	PGIV, NDUFA8	X	183‡	1–183	-Met, N-acetyl, 3 x Cys–Cys
**NUEM**	39 kDa, NDUFA9	P	384	8–268, 272–348, 355–375	NADPH
**NUXM**	—	O	192	1–192	N-acetyl
**NUJM**	B14.7, NDUFA11	Y	216	12–216	
**N7BM**	B17.2, NDUFA12	q	139‡	1–139	-Met, N-acetyl
**NB6M**	B16.6, NDUFA13	Z	146‡	1–141	-Met, N-acetyl
**ACPM1**	SDAPα, NDUFAB1α	T	138	46–138	4’-phosphopantethine + 3-hydroxytetradecanoate
**ACPM2**	SDAPβ, NDUFAB1β	U	130	43–130	
**NUTM**	—	f	86	2–79	
**NIGM**	AGGG, NDUFB2*	j	59	7–59	
**NB2M**	B12, NDUFB3	k	61	2–46	
**NB5M**	B15, NDUFB4	m	81	5–81	
**NUSM**	SGDH, NUNM, NDUFB5*	h	182	52–182	
**NUUM**	B17, NDUFB6†	i	74	4–72	
**NB8M**	B18, NDUFB7	o	87	2–81	Cys–Cys
**NIAM**	ASHI, NDUFB8	l	156	25–156	
**NI2M**	B22, NDUFB9	n	111	7–111	
**NIDM**	PDSW, NDUFB10	p	92	3–92	Cys–Cys
**NESM**	ESSS, NDUFB11	g	239	25–46,73–105, 132–234	
**NUQM**	—	1	190	21–189	
**NEBM**	B14.5b, NDUFC2*	d	78	1–75	

*Structural homologue, †positional homologue ‡-Met, +N-acetyl group.

Table S5 | Summary of the model built for state 4 of *Pp*-CI.

Subunit	Alternative names	Chain	Total residues	Modelled residues	Modelled cofactors and modifications
**NUAM**	75 kDa, NDUFS1	G	726	102–146, 160–188, 212–669, 681–704	2Fe2S, 2 x 4Fe4S
**NUCM**	49 kDa, NDUFS2	D	482	32–482	Dimethyl-Arg137
**NUGM**	30 kDa, NDUFS3	C	289	42–274	
**NUKM**	PSST, NDUFS7	B	204	30–204	4Fe4S
**NUIM**	TYKY, NDUFS8	I	222	31–222	2 x 4Fe4S
**NU1M**	ND1	H	353	1–353	N-formyl
**NU2M**	ND2	N	523	1–347, 365–523	N-formyl
**NU3M**	ND3	A	141	1–141	N-formyl
**NU4M**	ND4	M	491	1–491	N-formyl
**NULM**	ND4L	K	82	1–80	N-formyl
**NU5M**	ND5	L	642	1–642	N-formyl
**NU6M**	ND6	J	161	1–161	N-formyl
**NUYM**	18 kDa, NDUFS4	Q	159	35–60, 72–133	
**NIPM**	15 kDa, NDUFS5	e	106	2–106	2 x Cys–Cys
**NUMM**	13 kDa, NDUFS6	R	139	13–136	Zn^{2+}
**NIMM**	MWFE, NDUFA1	a	150	1–149	
**NI8M**	B8, NDUFA2	S	89‡	1–89	-Met, N-acetyl
**NI9M**	B9, NDUFA3	b	79	2–79	
**NUFM**	B13, NDUFA5	V	134	9–134	
**NB4M**	B14, NDUFA6	W	122	2–102, 109–122	
**NUZM**	B14.5a, NDUFA7	c	181‡	1–48, 70–103, 119–181	-Met, N-acetyl
**NUPM**	PGIV, NDUFA8	X	183‡	1–183	-Met, N-acetyl, 3 x Cys–Cys
**NUEM**	39 kDa, NDUFA9	P	384	8–375	NADPH
**NUXM**	—	O	192	1–192	N-acetyl
**NUJM**	B14.7, NDUFA11	Y	216	12–216	
**N7BM**	B17.2, NDUFA12	q	139‡	1–139	-Met, N-acetyl
**NB6M**	B16.6, NDUFA13	Z	146‡	1–141	-Met, N-acetyl
**ACPM1**	SDAPα, NDUFAB1α	T	138	44–138	4’-phosphopantethine + 3-hydroxytetradecanoate
**ACPM2**	SDAPβ, NDUFAB1β	U	130	43–130	
**NUTM**	—	f	86	2–79	
**NIGM**	AGGG, NDUFB2*	j	59	7–59	
**NB2M**	B12, NDUFB3	k	61	2–46	
**NB5M**	B15, NDUFB4	m	81	5–81	
**NUSM**	SGDH, NUNM, NDUFB5*	h	182	52–182	
**NUUM**	B17, NDUFB6†	i	74	4–72	
**NB8M**	B18, NDUFB7	o	87	2–81	Cys–Cys
**NIAM**	ASHI, NDUFB8	l	156	25–156	
**NI2M**	B22, NDUFB9	n	111	7–111	
**NIDM**	PDSW, NDUFB10	p	92	3–92	Cys–Cys
**NESM**	ESSS, NDUFB11	g	239	25–46,73–105, 132–234	
**NUQM**	—	1	190	21–189	
**NEBM**	B14.5b, NDUFC2*	d	78	1–75	

*Structural homologue, †positional homologue ‡-Met, +N-acetyl group.

Table S6 | Summary of key elements of the closed/active and open/deactive states from *Pp*-CI and mammalian CI.

Structural element	*Pp*-CI NUQM	*Pp*-CI NUQM-free	Active bovine [PDB: 8Q48]	Deactive bovine [PDB: 8Q49]
ND1-TMH5–6	Ordered	Ordered	Ordered	Disordered
ND1-TMH4	Bent (Tyr141 trigonal junction and Tyr156 in E-channel)	Bent (Tyr141 trigonal junction and Tyr156 flipped out of E-channel)	Bent (Tyr127 trigonal junction and Tyr142 in E-channel)	Straight (broken Tyr127 trigonal junction and Tyr142 flipped out of E-channel)
NDUFS2-β1- β 2	Ordered	Flexible (resolved but to a lower resolution than its surroundings)	Ordered	Disordered
ND3-TMH1–2	Ordered	Partially disordered and flexible (residues 58–61 not built, poorly resolved, Cys52 built)	Ordered	Disordered (residues 36–49 not built; Cys is residue 39)
ND6-TMH3	α -helix	π-bulge	α-helix	π-bulge
NDUFS7-Arg101	Pointing away from NDUFS2	Pointing away from NDUFS2	Pointing away from NDUFS2	Pointing towards NDUFS2

Table S7 | CryoEM data collection and refinement statistics for the proteoliposome dataset.

**Data collection and processing**				
Nominal magnification	73,000			
Voltage (kV)	200			
Electron exposure (e^{-} Å^{-2})	50.37			
Targeted defocus range (μm)	-1.8 to -3.3			
Calibrated pixel size (Å)	1.37			
Symmetry imposed	*C*1			
EMPIAR code	EMPIAR-12582			
Initial particle images (no.)	116,618			
State (EMDB code)	with flavoprotein (EMD-52893)	without flavoprotein (EMD-52892)	with NUQM (EMD-52891)	without NUQM (EMD-52890)
Final particle images (no.)	20,260	11,135	23,508	7,887
Map resolution (Å) (FSC 0.143)	4.7	19.7	4.7	8.2
Map sharpening (RELION) *B* factor (Å^2)	−96	−1301	−100	−268
